# Covid and social distancing with a heterogenous population

**DOI:** 10.1007/s00199-021-01377-2

**Published:** 2021-07-14

**Authors:** Miltiadis Makris

**Affiliations:** University of Kent, Canterbury, UK

**Keywords:** COVID-19, Epidemiology, SIR model, Social distancing, Equilibrium, Lockdowns, C61, C72, C73, H41, I12, I18

## Abstract

Motivated by the Covid-19 epidemic, we build a SIR model with private decisions on social distancing and population heterogeneity in terms of infection-induced fatality rates, and calibrate it to UK data to understand the quantitative importance of these assumptions. Compared to our model, the calibrated benchmark version with constant mean contact rate significantly over-predicts the mean contact rate, the death toll, herd immunity and prevalence peak. Instead, the calibrated counterfactual version with endogenous social distancing but no heterogeneity massively under-predicts these statistics. We use our calibrated model to understand how the impact of mitigating policies on the epidemic may depend on the *responses* these policies induce across the *various* population segments. We find that policies that shut down some of the essential sectors have a stronger impact on the death toll than on infections and herd immunity compared to policies that shut down non-essential sectors. Furthermore, there might not be an after-wave after policies that shut down some of the essential sectors are lifted. Restrictions on social distancing can generate welfare gains relative to the case of no intervention. Milder but longer restrictions on less essential activities might be better in terms of these welfare gains than stricter but shorter restrictions, whereas the opposite might be the case for restrictions on more essential activities. Finally, shutting down some of the more essential sectors might generate larger welfare gains than shutting down the less essential sectors.

**JEL Classification** C61 · C72 · C73 · H41 · I12 · I18

✉ Miltiadis Makris
mmakris.econ@gmail.com

### 1 Introduction

The current coronavirus epidemic has forcefully brought onto centre stage the need to understand how social distancing influences the evolution of an epidemic and vice versa. In the ongoing Covid-19 epidemic, infection-related deaths are more concentrated among older individuals and people with certain underlying health issues. Furthermore, even when faced with the same government policies on social distancing, individuals with less serious health consequences from infection seem to take more risks and social distance themselves less. This suggests that individuals have some form of discretion regarding their social interactions in the face of the same laws on social distancing. Motivated by these observations, in this paper we build and quantitatively implement a framework to model the interaction during an epidemic between private decisions on social distancing and government restrictions on socio-economic activity that slow the virus transmission, when (a) there is heterogeneity in the population regarding the risk of developing a serious illness when infected and eventually dying, and (b) governments cannot fully control social distancing of individuals.

Under imperfect control of socio-economic activity, individuals of different mortality-risks may respond differently to “mitigating” policies that restrict social interactions and economic activity. The reason is twofold. On the one hand, by influencing the evolution of infection, mitigating policies will affect the probability a susceptible person gets infected and thereby the scope for social distancing. On the other hand, private decisions on social distancing will naturally also depend, among others, on personal health conditions and any underlying health risk factors to develop a serious disease because of an infection. As a result, the impact of imperfect mitigating policies on the evolution of an epidemic and the welfare of the various risk-groups may actually depend on the *responses* these policies induce across the *various* segments of the population. Existing epidemiological models ignore this because they either treat social distancing as exogenous to the evolution of the epidemic, or assume that the government can control socio-economic activity and thereby social distancing. This paper makes a first attempt to fill this gap in the literature by building a simple fit-for-purpose variant of the basic SIR model (Kermack and McKendrick 1927; Anderson et al. 1992)—one of the most commonly used epidemiological models for the understanding of the current coronavirus epidemic. By doing so, we contribute to the improvement of our understanding of the welfare implications and impact on the evolution of an epidemic of imperfect mitigating policies.

Using existing estimates for the basic reproduction number, the length of the infectious period, and the length of the time from the onset of the infection to death, we calibrate our model to UK data on reported deaths during the current coronavirus epidemic to understand the quantitative significance for predictions of our novel assumption of endogenous social distancing by different mortality-risk groups.[1] Compared to our model, the calibrated benchmark version with exogenous and constant mean contact rate significantly over-predicts the mean contact rate over the duration of the epidemic, the death toll, exposure and the infection peak. Instead, the calibrated

[1] Similar data is available for several other countries and therefore our exercise could be applied to more countries as well.

counterfactual version with endogenous social distancing but with no heterogeneity in mortality rates massively under-predicts these statistics. We also use our calibrated model to study various hypothetical scenarios of imperfect government intervention on social distancing, and understand how the effects of various lockdown policies may depend on the behavioural responses these policies induce across a heterogenous population. The aim of our exercise is to highlight in the simplest possible manner how economics techniques could be integrated in epidemiological models to help us improve our understanding of the course of an epidemic and plan effective control strategies.

In general, social distancing refers to the adoption of a behaviour by individuals that reduces their risk of becoming infected. Examples are: limiting contact with other individuals or reducing the transmission risk during each contact. In our work, social distancing is modeled as a reduction in the proportion of all possible contacts a person can have. Social distancing incurs a cost in terms of isolation, distancing and alienating from friends and family, convenience, productivity, income e.t.c. As a result, individuals will only adopt (more) social distancing when there is a specific incentive to do so. Such incentives may include reducing the risk of death or the financial and health costs a person may face when infected. Typically, there is heterogeneity in the incentives to exert social distancing; for example, due to different infection-induced fatality rates across individuals with different health conditions. Our model allows us to study the different incentives to exert social distancing in the various segments of the population, and understand how these may change over the course of an epidemic. As it is known from the received literature, social distancing is increasing at the early stages of infection and decreasing at the later stages of infection. Typically, also, individuals that are of high risk to develop a serious illness when infected and eventually die will abstain more from social interactions than the rest of the population. Our model predicts that such high-risk individuals will exercise significant social distancing before any lockdown restrictions are even introduced, which is consistent with recent evidence.[2]

Our model predicts also that by the date of the first reported death, 0.009% of the UK population would have already been infected with the virus.[3] In addition, the epidemic wave in the UK would last around six months in the absence of government intervention. Third, our numerical analysis suggests that the peak of the mortality rate in UK would have occurred around mid April in the absence of government intervention, but with a significant death toll by the end of the epidemic.

We also find that the predicted evolution of the epidemic is significantly different quantitatively when social distancing is endogenous than when the mean contact rate is constant. Specifically, our simulations suggest that the death toll under endogenous social distancing is about one third of that under constant social distancing. Moreover, the mean contact rate over the duration of the epidemic is lower by about 10% compared to the case of constant social distancing. Furthermore, under endogenous social

[2] See Maloney and Taskin (2020). For related suggestive evidence based on UK mobility data from Apple, see https://www.independent.co.uk/news/uk/home-news/coronavirus-lockdown-uk-date-travel-a9516901.html.

[3] The corresponding number in Lourenço et al. (2020), where there is population heterogeneity in infection-induced mortality rates but the mean contact rate is assumed to be equal to one throughout the epidemic, is 0.08%.

distancing the predicted infection peak is less than half of, and the total exposure to the infection is a quarter less than, what is predicted under constant social distancing.

Crucially, social distancing decisions produce a positive externality as the higher the investment in social distancing the lower the rate of infection in the wider population is. Consequently, government intervention will typically be required in order to induce more social distancing. Motivated by this, our paper contributes also to the understanding of the impact of government intervention on social distancing and the epidemic by studying various lockdown episodes in terms of their welfare implications, their impact on the evolution of infection, exposure and death toll, and whether they are followed by a major after-wave or not. Importantly, the lockdown policies we focus on are not of first-best nature. That is, we do not investigate the solution to the optimal design problem that determines the optimal from the point of view of the society social distancing. The reason is that, despite its inherent theoretical interest, a full implementation of the optimal social distancing in large and modern societies would require massive administrative costs in terms of policing and of fine-tuning social interactions along several dimensions on a daily basis based on the state of the epidemic. We, therefore, choose to focus on "second-best" lockdown policies, which allow for some discretion on the part of individuals regarding social interactions.

Regarding the effects of various second-best government interventions on social distancing, our results suggest that the length of a lockdown that shuts down non-essential sectors such as services has a significant effect on the death toll, and the "flattening of the curve", but also on the behavioural responses of low-risk individuals when such policies are lifted. These behavioural responses, in turn, could contribute to an after-wave. These policies generate welfare gains compared to the case of no government intervention. Milder but longer restrictions on less essential activities might generate higher welfare gains than stricter but shorter restrictions.

Furthermore, our numerical analysis indicates that a government intervention that forces some of the essential sectors such as schools and parts of hospitality/entertainment industry to close down or reduce their operations has a significant negative impact on the cumulative count of deaths but not on the evolution of infections and herd immunity. The welfare gains from these intervention policies are large. Our numerical findings suggest that shutting down some of the more essential sectors generates much larger welfare gains than shutting down the less essential sectors. Moreover, stricter but shorter restrictions on more essential activities might generate much higher welfare gains than milder but longer restrictions. Our results suggest also that restrictions on the more essential sectors might not be followed by a second wave once these restrictions are lifted. The reason is that such mitigating policies will not affect social distancing by low-risk susceptible individuals during the lockdown.

To understand the latter finding, observe first that in the SIR model there is no after-wave when the mean contact rate times the proportion in the population of the susceptible to infection individuals times the basic reproduction number is (weakly) lower than one. This condition forms the basis of arguments in favour of strict lockdowns (a low mean contact rate) or of high herd immunity (a low pool of susceptible individuals) or of testing-and-tracing (a low basic reproduction number) in order to avoid a second wave of the infection. However, the mean contact rate is endogenous and depends on the evolution of the epidemic. Depending on the simulated lockdown policies, the equilibrium mean contact rate is roughly between 0.63 and 0.79 during the intervention period. With a basic reproduction number of 2.25 (used for instance in Lourenço et al. (2020) this implies that there will be no second wave when the simulated lockdowns are lifted, if the proportion in the population that has been exposed to the infection (and are no longer susceptible) is at least equal to around 44%. In our calibrated model, the equilibrium proportion of exposed people at the instant the simulated lockdowns are lifted is around 56%, and so there is no after-wave.

Furthermore, we identify a policy mix that consists of shutting some of the less essential and some of the more essential sectors on the day of the first UK lockdown, and leads to a similar death toll to the UK one at the time of writing these lines. We analyse the impact on the evolution of the epidemic, including the after-wave, that would emerge if this policy mix was lifted six weeks after its introduction. The result of this policy intervention is a significant flattening of the curve, followed by a sizeable second wave.

**Related Literature:** Our paper contributes to the literature that combines epidemiological models with equilibrium behavioral choice (for early contributions see Geoffard and Philipson 1996 and Fenichel 2013). We extend this literature by allowing for heterogenous mortality-risk groups. Compared to this literature we also discuss different types of policy interventions that de facto allow some discretion on the part of individuals regarding social interactions.

SIR models have been used extensively in mathematical epidemiology. Such models are particularly relevant for the study of the current SARS-CoV-2 epidemic because they assume that individuals become infectious as soon as they get infected and that recovered people do not get infectious again, which is a good first approximation of what is currently agreed by many experts to be the leading hypotheses for Covid-19. For, instance, it has been used in Lourenço et al. (2020), a study which found itself in the public spotlight.[4] In that paper, there is heterogeneity in infection-induced fatality rates. In addition, in the spirit of the traditional SIR model, social distancing is exogenously given. Our model, extends that work by (a) allowing for fatality rates of individuals who developed severe illness when infected to depend, due to capacity constraints in critical care provision, on the stock of infected people who become seriously ill, and (b) bringing private decisions on social distancing and equilibrium analysis at centre stage. Epidemiological models have also been used recently to discuss various issues on epidemics from a macroeconomics point of view.[5] In all these papers as well, individuals do not choose their social distancing.

Eichenbaum et al. (2020) and Krueger et al. (2020) instead study a macroeconomic version of the SIR model, where people choose to consume goods that require less social contact, and report that such behaviour can lead to a substantial mitigation of the economic and human costs of the current coronavirus crisis. Closer to our work is Reluga et al. (2010), and the independent works by Farboodi et al. (2020), Keppo et al. (2020) and Toxvaerd (2020), who analyse a SIR model with endogenous

4 "Coronavirus may have infected half of UK population—Oxford study", Financial Times, 24 March 2020, https://www.ft.com/content/5ff6469a-6dd8-11ea-89df-41bea055720b

5 See, for instance, Kaplan et al. (2020), Alvarez et al. (2020), Glover et al. (2020) and Bodenstein et al. (2020).

social distancing.[6] In contrast to our work, all these papers feature models with no heterogeneity in the population with respect to infection-induced mortality rates. Our work differs also from Reluga et al. (2010) and Toxvaerd (2020) in that they do not calibrate their models. Keppo et al. (2020) also discuss various lockdown policies, but, in contrast to our work, they assume that the cost from getting infected is constant over time. Moreover, in contrast to our work, Reluga et al. (2010) and Toxvaerd (2020) do not analyse government interventions regarding social distancing.[7] Keppo et al. (2020) and Farboodi et al. (2020) differ also from our work in that they assume that people do not know if they are infectious. Furthermore, when Farboodi et al. (2020) calibrate their model they assume that the mortality rate is constant, while we maintain throughout that the mortality rate of people who develop a serious illness from infection depends on the level of infections, thus capturing limited resources for critical care.[8]

The organisation of the paper is as follows. In the next section we develop the model, while in Sects. 3 and 4 we discuss equilibrium social distancing and welfare-improving government intervention on social distancing. In Sect. 5 we calibrate our model to UK data on reported deaths. There, we compare the predictions of our equilibrium model with those of a SIR model where social distancing is exogenous and with those of a SIR model where social distancing is endogenous but there is no heterogeneity in infection-induced mortality rates. We also study the implications for the evolution of the epidemic of various government interventions that induce higher social distancing than the equilibrium one in some parts of the population. Finally we conclude and point to possible extensions of our model and directions for future research.

### 2 The model

#### 2.1 The standard SIR model

We begin by setting up the standard SIR model. Let $N$ be the population size, which is assumed to be a very large number. We assume that the epidemic is fast relative to

[6] For more related literature, see the excellent discussion in Toxvaerd (2020). Galanis (2020) study in a static model the potential to use social proximity, and within-group random testing and anonymous notification of positive cases as a tool to induce social distancing.

[7] See, however, Rowthorn and Toxvaerd (2017) for a problem of managing an infection in an SIS model (where recovered can become infected again) with homogenous population and no risk of infection-induced mortality. Toxvaerd (2019) also study private social distancing and the planner's problem in a SIS model.

[8] Alvarez et al. (2020) calibrate their model of the optimal design of a lockdown within a SIR framework, in which, however, there is no heterogeneity in mortality rates and the government directly controls the contact rates in the population. Acemoglou et al. (2020) generalise Alvarez et al. (2020) by introducing heterogeneity between age groups with respect to infectiousness, need for critical care, mortality, and contact rates. They show that *optimal* policies differentially targeting age groups significantly outperform optimal uniform policies, and most of the gains can be realized by having stricter lockdown policies on the oldest group. These papers use data on both deaths and reported cases, while we use only data on deaths. The reason for our choice is that arguably reported cases are less accurate than reported deaths due to the lack of extensive testing and the existence of heterogeneity in intensity of symptoms that led to many mild cases going unreported, especially in UK. Moreover, while we focus on the first 14 days of the epidemic (based on reported deaths) to avoid contamination by mitigation strategies that were implemented after that time, Alvarez et al. (2020) use observations for the first 25 days after reported cases have been at least 100, and Acemoglou et al. (2020) use deaths reported on April 11 and cases reported 18 days earlier.

the demographic process, and so the population size $N$ is treated as constant. We also assume that individuals become infectious as soon as they get infected, a modeling assumption which seems to be roughly consistent with existing evidence for COVID-19. Denote by $t_0$ the instant when the epidemic starts (the “introduction time”).

Let $S$ be the proportion of the population that is susceptible to infection, $I$ the proportion of the population that is infected and infectious (or “prevalence”), and $R$ the proportion of the population that are no longer infectious. Denote also with $Z$ the proportion of the population that have been exposed to the infection and therefore are no longer available for infection.[9] That is,

$$Z = I + R = 1 - S \tag{1}$$

Let $\beta$ be the mean number of infections per unit of time if every person was susceptible and has been in contact with an infectious person. This is the rate of transmission of the infection, or the probability of an infection developing from a match between a susceptible and an infectious person. Denote with $\sigma$ the rate with which the typical infectious individual becomes non-infectious. Thus, $\frac{1}{\sigma}$ is the length of the infectious period (ie. the mean time until recovery or hospitalisation due to the development of a serious disease) per infectious individual. These two parameters are exogenous. We therefore have that the “basic reproduction number”, i.e. mean number of infections directly generated by one case if all individuals were susceptible and come in contact with the infected person, is equal to

$$\mathscr{R}_0 = \frac{\beta}{\sigma} \tag{2}$$

$\mathscr{R}_0$ measures the maximum reproductive potential for an infectious disease (at the beginning of the epidemic).

Note that under homogenous mixing of the population, $I$ is the probability with which a susceptible individual comes in contact with an infectious person. Allowing, instead, for non-homogenous but random matching, let $\gamma$ be the proportion of the infectious population with which a susceptible person comes in contact (or the probability with which a susceptible person comes in contact with an infectious person). We will refer to it as the mean contact rate. The two differential equations that determine $S$ and $I$ are thus:

$$\frac{dS}{dt} = -\beta\gamma SI \tag{3}$$

$$\frac{dI}{dt} = \beta\gamma SI - \sigma I \tag{4}$$

[9] Evidence so far indicates that for SARS-CoV-2, virtually all individuals who get infected are immune to future infection by the same strain, which supports our modeling assumption (when it comes to the SARS-CoV-2 epidemic) that susceptible, infectious and recovered individuals comprise the whole population.

with initial values $S(t_0) = S_0 \in (0, 1)$ and $I(t_0) = I_0 = 1 - S_0$. Note that (1) implies that

$$\frac{dZ}{dt} = \beta\gamma SI \tag{5}$$

with initial value $Z(t_0) = Z_0 = 1 - S_0 \in (0, 1)$. In this model, an epidemic with increasing number of infections initially starts when $\mathscr{R}_0\gamma S_0 > 1$, otherwise infections drop below their initial level from $t_0$ onwards and vanish over time. We will focus on the more interesting case where infections increase initially, noting however that in our model with endogenous social distancing the mean contact rate may be time-varying.

Hereafter, we will refer to $\beta\gamma S$ as the rate of infection, $\beta\gamma SI$ as the aggregate rate of infection, and $\beta\gamma S - \sigma$ as the spread of infection. Denote also with $\overline{I}(\gamma)$ the peak (i.e. the highest level) of the infection given the proportion $\gamma$ of the infectious population with which a susceptible person comes in contact. This is equal to[10]

$$\overline{I}(\gamma) = 1 - \frac{1}{\gamma\mathscr{R}_0}[1 + log(\gamma\mathscr{R}_0 S_0)]$$

We note here the dependence of the infection peak $\overline{I}(\gamma)$ on $\gamma$, which in general will depend on social distancing decisions. Therefore, the peak of prevalence will in general be different under endogenous social distancing decisions.

#### 2.2 The SIR model with heterogeneity in infection-induced mortality rates

The SIR model can also be extended to incorporate heterogeneity in infection-induced mortality rates at the population. Following Lourenço et al. (2020), we assume that there are two types of individuals. There are those who belong to a "high-risk" group (e.g. old people and people with asthma and other respiratory conditions e.t.c.), for whom getting infected leads with probability $\theta$ to a critical, and ultimately fatal, illness. We refer to these individuals as the high-risk individuals. Let $\psi$ denote the exogenously given delay between the time of infection and the time of death for a high-risk person who develops a critical illness from infection.[11] The number of the high-risk individuals is a proportion $\rho$ of the population. The rest of the population, hereafter referred to as the low-risk individuals, will not die when infected. We will indicate risk-types with the index $i = L, H$, where $L$ stands for the low-risk and $H$

[10] In the standard SIR model we have that $S_t = S_0 e^{-\frac{\beta\gamma}{\sigma}(S_0 + I_0 - S_t - I_t)}$. The infection peak occurs when $S_t = \frac{\sigma}{\beta\gamma}$. using this in the previous equation together with $S_0 + I_0 = 1$ and the definition of $\mathscr{R}_0$ gives the condition for $\overline{I}(\gamma)$.

[11] An alternative modeling choice would be to assume that high-risk individuals who develop a critical illness from infection die at a constant rate. We have, however, chosen to maintain the above modeling assumption in Lourenço et al. (2020) of an exogenous delay between the time of infection and the time of death to facilitate comparability and highlight the implications of introducing endogenous social distancing in an established epidemiological model for the understanding of the Covid-19 crisis.

for the high-risk type.[12] Denote the cumulative count of deceased $\psi$ periods ahead as a proportion of the population by $\Lambda$.

We extend the model in Lourenço et al. (2020) in two ways. First, by assuming that the mortality rate $\theta$ is increasing with the share in the population of the infectious individuals who become seriously ill when infected, i.e.

$$\theta = F(\rho I)$$

where $F : [0, 1] \to (0, 1)$ is a (weakly) increasing function such that $F(0) \in (0, 1)$. This assumption captures an environment where resources for effective critical health care are limited and thereby health services get stretched (and even overwhelmed) when the demands for intensive care are high.[13] We also assume that $F$ is convex for low levels of $\rho I$ to capture capacity constraints (such as a limited number of ICUs) in a smooth way.[14] This assumption is plausible in the context of the ongoing coronavirus epidemic, and therefore important for the quality of the predictions of the calibrated model. This assumption implies also that the cost of high-risk susceptible individuals becoming infected is more increasing in the number of infected people than the corresponding cost of low-risk individuals. This will be an additional source of heterogeneity in the social distancing decisions of individuals and thereby in the impact of imperfect mitigating policies on the different segments of the population.

We further extend the model in Lourenço et al. (2020) by allowing for different probabilities of coming in contact with an infectious person for high-risk and for low-risk individuals. Denote with $n^i$ the probability with which a susceptible person of risk-type $i = L, H$ comes in contact with an infectious person. We then have that

$$\frac{d\Lambda}{dt} = F(\rho I)\rho n^H \beta S I \tag{6}$$

with $\Lambda(t_0) = F(\rho I_0)\rho I_0$. To understand this law-of-motion, we note that $\rho\beta S I$ represents the new infections (as a proportion of the population) in the current instant among the high-risk susceptible individuals, had this type of individuals been in contact with all the infectious people in the population. However, high-risk susceptible individuals come in contact with other people at a rate $n^H$. Therefore, the proportion of infected high-risk individuals in the population is $\rho n^H \beta S I$, and hence $F(\rho I)\rho n^H \beta S I$ is the increase in deaths (as a proportion of the population) in $\psi$ periods ahead, all coming from the part of the susceptible population which is of high risk. Note that when every individual meets with everyone else (as in Lourenço et al. 2020), we have that $n^H = 1$

[12] Evidence so far indicates that for SARS-CoV-2, the vast majority of infected individuals who die have had underlying health problems that were exacerbated due to the infection, and so our assumption that low-risk individuals do not die due to the infection seems to be a good modeling approximation of the SARS-CoV-2 epidemic.

[13] Therefore, our model can also be used to study government interventions whereby more resources are channelled to health care services and as a result $F(.)$ decreases for any given $\rho I$. However, as our focus is on social distancing, we leave such an analysis for another paper.

[14] See, for instance, Kaplan et al. (2020) and Glover et al. (2020) for alternative ways to introduce capacity constraints that allow for a kink at the point when the capacity constraint binds.

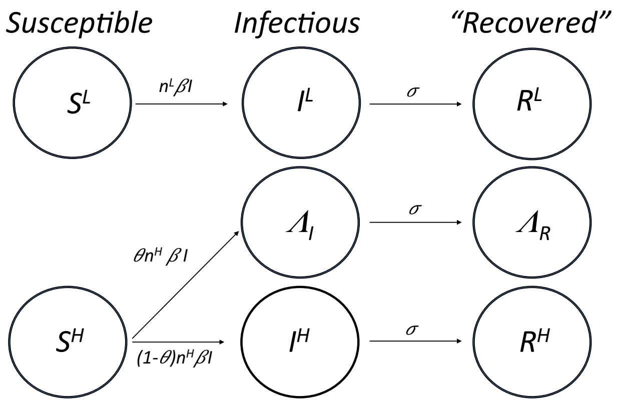

**Fig. 1** States and Flows in the SIR model with heterogenous infection-induced mortality rates and $\gamma = 1$. Here, however, due to endogenous social distancing, we may have that in equilibrium $n^H < 1$ and, thereby, $\gamma < 1$. We return to this shortly.

In this version of the SIR model (see Fig. 1), the pool $S$ includes all the $(1-\rho)S$ low-risk susceptible individuals ($S^L$) and all the $\rho S$ high-risk susceptible individuals ($S^H$); the pool $I$ includes all the $(1-\rho)I$ low-risk individuals ($I^L$) who become infectious (at a rate $n^L\beta$), all the $(1-\theta)\rho I$ high-risk individuals who become infectious (at a rate $(1-\theta)n^H\beta$) but survive the infection ($I^H$), as well as the $\theta\rho I$ high-risk individuals who become infectious (at a rate $\theta n^H\beta$) but become terminally ill and die in $\psi$ periods ($\Lambda_I$). Moreover, the pool $R$ includes all the $(1-\rho)R$ low-risk individuals ($R^L$) who recover (at a rate $\sigma$), the $(1-\theta)\rho R$ high-risk individuals ($R^H$) who survive the infection and recover (at a rate $\sigma$) as well as the $\theta\rho R$ high-risk individuals who become terminally ill and die in $\psi$ periods but become non-infectious (at a rate $\sigma$) due to hospitalisation ($\Lambda_R$). All the high risk-individuals who became infected and will die in $\psi$ periods are in the pool $\Lambda = \Lambda_I + \Lambda_R$, whether they are infectious or not. More details on this version of the SIR model are given in the "Appendix".

The above model could be extended in a number of ways to allow for further heterogeneity in the population. For instance there could be heterogeneity in the transmission rates based on the risk type, or in the recovery rates based on the risk type and whether an individual has developed a critical illness.[15] There could also be heterogeneity within the group of high-risk individuals regarding the delay from the time of the onset of the critical illness to the time of death. We choose to abstain from such heterogeneities for two reasons. First, there is not enough evidence on the degree of such heterogeneities that we can use to guide our modeling assumptions and associated calibration. Therefore, we choose to be agnostic on such heterogeneities and focus only on the heterogeneity with respect to infection-induced mortality as in Lourenço et al. (2020). Second, we want to focus on understanding how the novel economic part of our model – endogenous social distancing by different mortality-risk groups – affects the predictions of the SIR model and its related variants, and so we choose to keep the

[15] See Acemoglou et al. (2020) for a model with such heterogeneity, but where individuals do not choose their own social distancing.

details of the epidemiological model as simple as possible to facilitate comparability. Introducing more heterogeneity would be interesting from a theoretical point of view and might also improve the overall predictive power of the model, but would not allow us to highlight the added value of our contribution in a clear and stark manner.

We leave this section by defining the "effective reproduction number" as $\gamma \times \mathscr{R}_0 \times S$. This is the number of infections produced by an infectious person. Recalling (4) and using the definition of $\mathscr{R}_0$, we note that if the effective reproduction number is greater than one, then infections will grow given the prevailing herd immunity, $1 - S$. As infections grow, the pool of susceptible individuals becomes smaller, the effective reproduction number eventually drops below one and infections start decreasing, vanishing towards the end of the epidemic.

#### 2.3 Incorporating social distancing in the SIR model

In our model, we also allow for endogenous social distancing. Each individual is assumed to be a recipient of a flow utility which depends on her/his risk-type and the state she/he is at. The states are Susceptible, Infected and Recovered. The expected utility also depends on the probability of getting infected, which, in turn, depends on own social distancing choices, the social distancing choices of the rest of the population and prevalence.

Given any state, the flow utilities are (weakly) higher for low-risk individuals. Given any risk type, the flow utility in the Susceptible state is (weakly) higher than the flow utility in the Recovered state, and the flow utility in the latter state is (weakly) higher than the flow utility in the Infected state. We assume that risk- and state-dependent flow utilities are constant.[16] The transition rate between the Susceptible state and the Infected state for any individual is equal to $\beta S$ times the individual's contact rate. The latter is (partly) controlled by the given individual.

Specifically, every individual pays a cost associated with social distancing in exchange for a reduction in their own risk of becoming infected. We restrict attention to pure strategies. Each individual is very small relative to the rest of the population. Therefore, each individual takes the levels of infection and susceptibility, $S$ and $I$, and their evolution, to be exogenously given and out of their control. Individuals take also as given and outside their control the mean contact rate in the population. In equilibrium, the anticipated (state-dependent) mean contact rate in the population is equal to the actual mean contact rate implied by individuals' equilibrium decisions.

[16] In principle, the flow economic costs faced by infected individuals may not be time-invariant. This would, for instance, be the case in the presence of imperfect capital markets. For our paper, we implicitly assume that there are no capital market imperfections. We also assume that the economy is at a steady state when it is hit by the epidemic, and that the proportion of the government budget devoted to the support of the economy during adverse environmental/health aggregate shocks is sufficient for insulating the economy from any medium- and long-run consequences of such shocks. Assuming that the epidemic is fast relative to the evolution of the economy allows us then to treat the income earned and consumption under each state as constant over the duration of the epidemic. As a result, the flow utilities can be treated as constant over time. An interesting extension of our model, which we leave for future work, would be to study the implications for social distancing, and hence the evolution of the epidemic, of various capital market imperfections and limitations in the ability of governments to borrow/tax to insulate the economy.

Following the existing literature, we assume that any societal motivation for social distancing is small relative to the marginal cost of exercising social distancing. A direct implication of this assumption is that infectious individuals do not exercise social distancing, and thus the burden of social distancing falls with the non-exposed individuals. Given this assumption, and in order to simplify notation, we assume hereafter that there is no societal motivation for social distancing. We also assume that the instance of becoming infectious coincides with the onset of symptoms. As a result, no infectious individual mistakenly behaves like a non-exposed one. While recognising that both these assumptions may be restrictive,[17] we also note that relaxing them might reduce further the equilibrium rate of infection and make it potentially more responsive to second-best lockdown policies.[18] Therefore, despite the fact that relaxing these two assumptions might potentially generate new insights in addition to those our paper produces, the main message of our work might even be strengthened after relaxing these two assumptions. We leave such an extension for future work.

We also assume that individuals know their risk type (i.e. whether they are old or have underlying respiratory problems). In reality, there can be cases where some people do not know whether they are of low- or high-risk type.[19] However, we conjecture that this type of individuals will only be a very small percentage in the population, with most people having a strong indication of whether they have an underlying condition that would classify them as high-risk types in our model's lingo. Therefore, ignoring this group of individuals might not be with much loss of generality.

Except their risk-type, individuals are ex ante identical to each other in any other aspect. We thus focus on pure-strategy equilibria where all susceptible individuals of the same risk-type choose the same pure-strategy social distancing.

We formulate the social distancing problem of each non-exposed (i.e. being at the Susceptible state) individual as one where she/he chooses (through altering socio-economic behaviour) the probability with which she/he severs a contact (or,

[17] Similar assumptions are used in Reluga (2010) and Toxvaerd (2020).

[18] An individual who is infectious but has no symptoms yet may attach a positive probability on them being susceptible. As a result, they may also adopt some social distancing, which, in turn, will reduce the probability a susceptible person will come in contact with an infectious person, all other things equal. A sufficiently high societal motive on the part of infectious individuals will induce them to adopt some social distancing, which, in turn, will also reduce the probability a susceptible person will come in contact with an infectious person, all other things equal. Both these effects operate towards reducing the equilibrium rate of infection. However, other things may not stay the same. The reason is that non-infectious individuals might adopt a lower level of social distancing because (a) a lower probability with which they will come in contact with an infectious person (due to infectious individuals exercising some social distancing) implies a lower risk of getting infected, and (b) susceptible individuals may attach a positive probability on them already being infected but without symptoms and thereby exercise less social distancing compared to the case when the instance of becoming infectious coincides with the onset of symptoms. Therefore, whether the equilibrium rate of infection is lower or not as a result of allowing for societal motive and for pre-symptomatic infection will eventually depend on the relative strength of the direct effect on infectious individuals' social distancing and the indirect—equilibrium—effect on susceptible individuals' social distancing. If the direct effect is stronger, then the net effect of introducing a societal motive or pre-symptomatic infection will be to reduce the equilibrium rate of infection.

[19] Uncertainty over own risk type would make susceptible individuals exerting more social distancing than the low-risk individuals in our model, but less social distancing than the high-risk individuals in our model.

equivalently, the proportion of all the contacts she/he severs).[20] We denote by $\delta_i$ the percentage of potential contacts a susceptible individual of risk type $i = L, H$, severs by exercising social distancing. That is, the contact rate of individuals of risk type $i$ is equal to $n^i = 1 - \delta_i$.

We postulate that the costs of reducing more essential socio-economic activities are higher than the costs of reducing less essential socio-economic activities. As a result, when an individual wants to increase their socially distancing, they will do so by reducing first the least essential socio-economic activities from the available activities. This assumption implies an environment where, starting from two different degrees of social distancing, a small increase in social distancing is associated with reducing more essential social interactions and economic activities in the higher of the two starting degrees of social distancing; that is, an environment with convex costs from social distancing. To simplify the analysis, we restrict attention to quadratic costs.[21]

We also postulate that social distancing may face exogenous bounds $0 \leq \underline{\delta} \leq \overline{\delta} \leq 1$. For instance, $\underline{\delta} > 0$ can capture a government intervention that enforces a minimum level of distancing such as a curfew. $\overline{\delta} < 1$, on the other hand, may be the case because people may need to interact with other people from their network for essential shopping, getting help, catering for their pets or other family members in need etc. Related, some people may have to go to work either because they are essential workers or because they face pressure from employers. The latter raises another avenue for government intervention towards enabling more social distancing. We will refer to $\underline{\delta}$ as the minimal (degree of) social distancing, and to $\overline{\delta}$ as the maximal (degree of) social distancing.

Let $V_I^i$ be the (expected) net present value (NPV) of being infectious and of risk-type $i = L, H$ but surviving the infection (which happens with probability one for low-risk individuals and probability $1 - F(\rho I)$ for high-risk individuals.) Given our assumption that flow utilities are constant, and that the Recovered state is an absorbing state, this NPV is also constant. Let also $V_S^i$ be the NPV of being susceptible to infection and of risk-type $i = L, H$. Denote with $K_i$ the expected net present value of the welfare cost of susceptible individuals of risk-type $i = L, H$, from getting infected and surviving

[20] For an investment towards social distancing $c$, we have that only $d(c)$ proportion of feasible contacts survive, where $d(c)$ is a decreasing and (weakly) convex function (see also Reluga et al. 2010). Denoting with $\delta$, the proportion of feasible contacts a person severs, and setting $1 - \delta = d(c)$ allows us to define the cost of inducing a reduction $\delta$ in the probability of a contact taking place as $d^{-1}(1 - \delta)$. For instance, if $d(c) = \frac{1}{1+mc}$, $m > 0$, then the cost of inducing a reduction equal to $\delta$ is $m^{-1}\frac{\delta}{1-\delta}$. If, as another example, $d(c) = 1 - \sqrt{2mc}$, $m > 0$, then the cost of inducing a reduction equal to $\delta$ is $m^{-1}\frac{\delta^2}{2}$. As a final example, if $d(c) = 1 - \frac{c}{m}$, $m > 0$, then the cost of inducing a reduction equal to $\delta$ is $m\delta$.

[21] An alternative assumption that has appeared in the literature is that of a constant marginal cost from social distancing, which presumes uniform costs from social distancing across all activities. Under such an assumption private social distancing takes the form of a step function. To be more precise, if the expected health cost (given current infectiousness) under no social distancing is below the constant marginal cost from exercising social distancing, then private social distancing is equal to its lowest feasible level, while if the expected health cost under no social distancing is above the constant marginal cost from exercising social distancing, then private social distancing is equal to its highest feasible level (see, for instance, Toxvaerd 2020.)

the infection, i.e.

$$K_i = V_S^i - V_I^i$$

We assume hereafter that the difference between the flow utilities of low-risk people and the flow utilities of high-risk individuals under the Infected and under the Recovered state are high enough so that $K_H > K_L$. In addition to the cost from getting infected but surviving the infection, high-risk individuals face also an additional cost from becoming critically ill and eventually dying ($\psi$ periods ahead), denoted by $\overline{K}$ in present value terms, and assumed to be positive. We show in the "Appendix" that, given our assumptions, this cost is constant. We assume that the cost from dying, $\overline{K}$, is finite.[22] Finally, denote with $C_i$ the expected net present value of the welfare cost faced by a susceptible individual of risk-type $i = L, H$, from getting infected in the next instant, i.e.

$$C_L = K_L$$

and

$$C_H = K_H + F(\rho I)\overline{K}$$

Given these definitions, we can show that in equilibrium $K_i$ is determined by the following law-of-motion:

$$\frac{\partial K_i}{\partial t} = \phi^i + \xi K_i + [1 - \delta_i]\beta I C_i + \frac{{\delta_i}^2}{2}, \tag{7}$$

where $\xi$ denotes the discount rate, and $\phi^i$ is a constant which depends on the discount rate, the recovery rate, and the flow utilities for an individual of risk-type $i = L, H$, when susceptible, when infectious but surviving the infection, and when recovered. The above law-of-motion is, in effect, the formula that gives the equilibrium NPV from being at the Susceptible state of an individual of risk-type $i = L, H$, $V_S^i$, after using that $K_i = V_S^i - V_I^i$ and rearranging (see the "Appendix" for more details.)

We show in the "Appendix" that every non-exposed individual of risk-type $i = L, H$, solves, in effect, the following problem:

$$\max_{\delta_i \in [\underline{\delta}, \overline{\delta}]} \delta_i \beta I C_i - \frac{\delta_i^2}{2}$$

where we note that $\delta_i \beta I$ is the reduction in the probability of getting infected in the next instant given current social distancing $\delta_i$. All individuals take $\beta I C_i$ to be outside their control. Note thus that the optimal social distancing of an individual of risk-type

[22] For a recent treatment of the economic cost of death see Kniesner and Viscusi (2020). For a collection of estimates of the *economic* value of life used in various cost-benefit policy studies see https://en.wikipedia.org/wiki/Value_of_life.

$i = L, H$, minimises the right-hand size of the above law-of-motion (7), i.e. the rate of increase of the cost from getting infected but surviving the infection.

It follows that in equilibrium every susceptible low-risk individual chooses social distancing

$$\delta_L = \delta_L^*(K_L, I) \equiv \max\{\underline{\delta}, \min\{\beta I K_L, \overline{\delta}\}\} \tag{8}$$

To understand this note that $\delta_L$ is the marginal cost and $\beta I K_L$ is the marginal benefit from social distancing. The marginal benefit and, thereby, the chosen social distancing by low-risk individuals is, as intuition would suggest, increasing in the infection rate $\beta I$ and the cost of getting infected, $K_L$.

In turn, in equilibrium, every susceptible high-risk individual chooses social distancing

$$\delta_H = \delta_H^*(K_H, I) \equiv \max\{\underline{\delta}, \min\{\beta I[K_H + F(\rho I)\overline{K}], \overline{\delta}\}\} \tag{9}$$

for any given level of infection $I$. To understand this recall that $\delta_H$ is the marginal cost and note that $\beta I[K_H + F(\rho I)\overline{K}]$ is the marginal benefit from social distancing. The marginal benefit and, thereby, the social distancing by high-risk individuals is, as intuition would suggest, increasing in the transmission rate $\beta$, prevalence $I$, the cost of getting infected but surviving the infection $K_H$, and the cost of dying $\overline{K}$. Observe that

$$\delta_H^*(K_H, I) \geq \delta_L^*(K_L, I)$$

due to $F(\rho I) \geq F(0) > 0$, $K_H > K_L$ and $\overline{K} > 0$.

Furthermore, we have that in equilibrium:

$$n^i = 1 - \delta_i^*(K_i, I)$$

for any $i = L, H$, and

$$\gamma = \gamma(K_L, K_H, I) \equiv (1-\rho)\left(1 - \delta_L^*(K_L, I)\right) + \rho(1 - \delta_H^*(K_H, I))$$

Clearly, then, the evolution of the equilibrium social distancing will depend on the evolution of the epidemic as well as the evolution of the costs from getting infected and surviving the infection $K_i$.

Before we leave this section, we note that the above decision problem could potentially be extended in several ways. For instance, there could be heterogeneity with respect to the cost of social distancing; high-risk individuals might face a lower cost of social distancing if they are retired or are under care. Such a model would lead to even lower social distancing for the group with the higher cost from social distancing compared to the group with the lower cost from social distancing. There could also be heterogeneity in terms of the maximal social distancing people can exercise; high-risk individuals might face a higher upper bound because they may not work anyway or because they have arranged care. Such a model could lead to even higher social distancing on the part of high-risk individuals.[23] Moreover, observe that the above problem would also describe individual decision-making in a population where people do not know their risk type, but the population consists of two groups with different beliefs about the likelihood that they are of high or low risk. In such a model, the subscript $i$ would indicate the group in terms of these beliefs and $C_i$ would also take into account the uncertainty from the point of view of individuals about their risk type. We choose to abstain from such very interesting and plausible types of (additional) heterogeneity, as well as from other possible extensions (see the Conclusions), because we want to focus on understanding how the novel part of our model affects the predictions of the SIR model and its related variants. Therefore, we choose to keep the details of the economic part of our model as simple as possible in order to facilitate comparability, and emphasise our contribution in a clear and focused manner. By doing so, we hope to make the case for the scope of future research that constructs even more elaborate models with potentially higher predictive power.

### 3 The equilibrium SIR model

In equilibrium, the two differential equations above that determine $Z$ and $I$ become:

$$\frac{dZ}{dt} = \left[(1-\rho)\left(1-\delta_L^*(K_L, I)\right) + \rho(1-\delta_H^*(K_H, I)\right]\beta(1-Z)I \tag{10}$$

$$\frac{dI}{dt} = \left\{\left[(1-\rho)\left(1-\delta_L^*(K_L, I)\right)+\rho(1-\delta_H^*(K_H, I))\right]\beta(1-Z)-\sigma\right\} I \tag{11}$$

with initial values $Z(t_0) = I_0$ and $I(t_0) = I_0 \in (0, 1)$.

In addition, we have that the law-of-motion for the cumulative number of deaths at time $t + \psi$ is equal to

$$\frac{d\Lambda}{dt} = F(\rho I)\rho(1-\delta_H^*(K_H, I))\beta(1-Z)I \tag{12}$$

with initial value

$$\Lambda(t_0) = F(\rho I_0)\rho I_0$$

This law-of-motion will be used for the calibration of our model to UK data on reported deaths.

Finally, the laws-of-motion above that govern the evolution over time of the costs from getting infected but surviving the infection for the low- and high-risk individuals

[23] Such a model would also allow the study of "targeted" second-best lockdown policies such as those restricting socio-economic activities of high-risk individuals only. See Acemoglou et al. (2020) for a study of targeted first-best lockdown policies, i.e. under the assumption that the planner can control directly the social distancing of different groups.

become

$$\frac{\partial K_L}{\partial t} = \phi^L + \xi K_L + [1 - \delta_L^*(K_L, I)]\beta I K_L + \frac{(\delta_L^*(K_L, I))^2}{2} \tag{13}$$

and

$$\frac{\partial K_H}{\partial t} = \phi^H + \xi K_H + [1 - \delta_H^*(K_H, I)]\beta I[K_H + F(\rho I)\overline{K}] + \frac{(\delta_H^*(K_H, I))^2}{2}, \tag{14}$$

respectively. Let $K_{i0}, i = L, H$, denote the initial costs from getting infected and surviving the infection. These are endogenously determined to ensure convergence to a steady state.

Note that, with $\underline{\delta} = 0$, $I_0 > 0$ and $S_0 > \frac{1}{\gamma(K_{L0}, K_{H0}, I_0)\mathscr{R}_0}$, in any steady state with zero prevalence and with finite costs from being infected and surviving the infection, denoted by $K_L^*$ and $K_H^*$, we have zero social distancing from all individuals. Consequently, in any such steady state, we have $K_i^* = \frac{-\phi^i}{\xi}$ for all $i = L, H$. In such equilibrium, the pool of infected increases over time until it reaches an endogenously determined peak at which time the number of exposed individuals is high enough for the spread of infection to become negative and infections start decreasing all the way to zero. Interestingly, we note that infections will peak quicker than in the absence of social distancing as now an increase in infections raises social distancing as well (at least initially) reinforcing the standard effect on the rate of infection of a lower pool of susceptible people. Similarly when $\underline{\delta} > 0$; the only difference in this case is that the steady-state values of the costs from getting infected but surviving the infections are appropriately modified to ensure that $\frac{\partial K_i}{\partial t} = 0$ for all $i = L, H$.

Unfortunately, deriving an analytic solution for the system of highly non-linear ODEs (10)-(14) is very difficult and we have to resort to numerical solutions. These solutions will depend on the parameter choices. Existing estimates and the calibration of our model to data on reported coronavirus-related deaths in UK will guide our selection of the model parameters. Before we do so in Sect. 5, we discuss next various scenarios of government intervention.

### 4 Government intervention

Private decisions on social distancing produce an externality due to the fact that individuals do not internalise that their social distancing decisions have an effect on the mean contact rate and thereby on the evolution of the epidemic over time. Therefore, individuals do not take into account that an increase in their social distancing reduces the future risk of infections and future probability of death faced by other individuals. This externality is positive, and will be the dominant one if the cost from dying $\overline{K}$ is sufficiently high. In such an environment, the optimal (from the point of view of a Utilitarian planner) social distancing is (weakly) higher than that chosen by susceptible individuals themselves. See the “Appendix” for the details.

Importantly, despite its inherent theoretical interest, a full implementation of the optimal social distancing in large and modern societies would require massive administrative costs in terms of policing and fine-tuning social interactions along several dimensions on a daily basis. In fact, anecdotal evidence and news items during the current coronavirus crisis suggest that, even when faced with the same government policies on social distancing, low-risk individuals seem to take more risks and social distance themselves less. This suggests that individuals have some discretion regarding their social interactions in the face of the same laws on social distancing. We, therefore, choose to focus hereafter on “second-best” lockdown policies and their effects on the evolution of the epidemic. There are potentially several ways to model second-best lockdown policies, depending on the specific assumptions about the enforcement of social distancing restrictions. In this paper, we will focus only on a few of these modeling options.

Despite its inability to fine-tune social distancing along several dimensions on a daily basis given the state of the epidemic, the government can still impose certain restrictions on socio-economic activities. For example, it can shut down less essential sectors, or limit their operations, impose a curfew or restrict large gatherings. In terms of our model, such policies will force individuals to refrain from the less essential activities. Given our interpretation of higher degrees of social distancing as refraining from more essential activities, such policies can thus be captured in our model by an increase in the minimal degree of social distancing $\underline{\delta}$.

Governments can also introduce strict social distancing restrictions in some of the essential sectors such as schools, universities, government buildings and agencies. They can also provide food delivery and in-house care to high-risk individuals.[24] In terms of our model, such policies will enable individuals to refrain from some of the more essential activities. Given our interpretation of higher degrees of social distancing as staying away from more essential activities, such policies can thus be captured in our model by an increase in the maximal degree of social distancing $\overline{\delta}$.

At this stage, the following remarks are in order.

#### 4.1 Two remarks on the modeling of second-best policies

Some readers may argue that the way policy is modeled in the paper seems to depend significantly on what cost function is employed. We note that this is not the case insofar one restricts attention to convex cost functions, as we do here. The reason is the following. A linear cost function leads always to corner solutions, which might make it not the best modeling choice, especially if one wants to describe what we observe in reality, where we see many people taking risks with some people taking more risks than others. On the other hand, a convex cost function (including the quadratic one we use here) does allow for interior solutions in principle: interior social distancing will take place as long as the marginal benefit is in some appropriately defined intermediate

[24] In reality, the costs of such provisions may be covered through higher current public debt and higher taxation in the future. In our model, these costs are encompassed by the social-distancing cost function.

range. Importantly, thus, it is the boundary conditions on social distancing that are in operation when it comes to forcing corner solutions: the minimal degree of social distancing ($\underline{\delta} \geq 0$ in our model, $\underline{\delta} = 0$ in other models in the literature) for low-risk individuals, and the maximal degree of social distancing ($\overline{\delta} \leq 1$ in our model, $\overline{\delta} = 1$ in other models in the literature) for high-risk individuals. In fact, such boundary constraints, set at the appropriate level, would force corner solutions for *any* convex cost function. At this stage some readers may argue that relatively small changes in the boundaries $\underline{\delta}$ and $\overline{\delta}$ would not affect agents' behavior. Would we then just say these agents are not responsive to policy? True, small changes of the boundary constraints will not change interior behaviour, but large changes will, and policies as conceptualized in this paper could induce large changes in the boundary degrees of social distancing: some extreme examples are a strict curfew, or shutting down the whole economy except hospitals.

Given the justification in Sect. 2.3 on the use of a convex cost function, thinking about alternative ways of modeling second-best policy stumbles on the issue of costly design and enforceability. The reason is that any attempt to affect interior social distancing decisions in ways different than those envisaged here (such as stay-at-home a certain level or percentage more than what would have been the case without government intervention) would also require high design and implementation costs. To be consistent with the narrative of the paper on enforceability of policies, we therefore chose to not discuss other (more demanding in terms of enforcement) policies.

In the remainder of this section, we discuss the qualitative effects of the policies we focus on.

#### 4.2 Reducing more essential activities

High-risk individuals may face a high probability of dying after getting infected, with this probability being increasing with infections due to capacity constraints in critical care provision. As a result, high-risk individuals are more likely than low-risk individuals to choose the maximal level of social distancing, severing all but essential contacts. Thus, one way with which the government could intervene and reduce deaths would be to shut down some of the essential sectors of the economy or reduce their operations, thereby allowing high-risk individuals to insulate themselves more. An example of this is the government providing the option of in-house medical care/support and groceries delivered at home, with the costs ultimately passed on to individuals (through a direct charge or future taxation). In our model, this can be captured by an exogenous increase in maximal social distancing $\overline{\delta}$.

An increase in maximal social distancing $\overline{\delta}$ is more likely to decrease (at the later stages of the epidemic) the rate of infection among the high-risk individuals, $\rho(1 - (\delta^*_H(K_H, I))\beta(1 - Z)$, for any given *infection capacity* $\beta(1 - Z)$, than the rate of infection among the low-risk individuals. This has a number of implications. First, the decrease in the rate of infection among high-risk individuals reduces the number of future deaths (recall (12)). This reduction comes both through a direct reduction in the infection rate among high-risk individuals (for given fatality rate) and through a reduction in the future mortality rates (for given future infection rates among highrisk individuals). These effects emphasise the importance of government interventions that increase social distancing from high-risk people in terms of reducing the count of fatalities. Second, the decrease in the rate of infection due to an increase in $\overline{\delta}$ reduces the flow of infection $\frac{dI}{dt}$, but it also reduces the flow of exposure to the infection $\frac{dZ}{dt}$ and thereby the dampening effects of new infections on the future flow of infections (i.e. the $1 - Z$ term in (11) after recalling (10)). Therefore, increasing social distancing from high-risk individuals has an ambiguous effect on the *duration* of the infection, with the net effect depending on the primitive factors of the infection such as the relative sizes of $\rho$, $\beta$ and $\sigma$.

#### 4.3 Restricting less essential activities

Shutting down or reducing the operations of the less essential sectors of the economy such as restaurants, banning large gatherings, or imposing a curfew is more likely to increase social distancing of low-risk individuals than social distancing of high-risk individuals. Thus, one way with which the government could intervene and increase social distancing in the early stages of the epidemic would be to restrict the least essential activities. In our model, this can be captured by an exogenous increase in minimal social distancing $\underline{\delta}$.

An increase in minimal social distancing $\underline{\delta}$ is more likely to decrease (at the early stages of the epidemic) the rate of infection among low-risk individuals, $(1-\rho)(1-(\delta_L^*(K_L, I))\beta$, than the rate of infection of high-risk individuals.[25] This has a number of consequences. First, it reduces the number of future deaths. This reduction comes indirectly through the lowering of the mean contact rate and thereby the relief of health services and the associated reduction in the mortality rates among high-risk individuals in the future. This novel effect highlights the significance of government interventions that increase social distancing of low-risk individuals in terms of reducing the count of fatalities. Second, the decrease in the rate of infection due to the increase in social distancing of low-risk individuals reduces the flow of infection $\frac{dI}{dt}$, but it also reduces the flow of exposure to the infection $\frac{dZ}{dt}$ and thereby the dampening effects of new infections on the future flow of infections. Therefore, increasing social distancing of low-risk individuals also has an ambiguous effect on the *duration* of the infection, with the net effect depending on the primitive factors of the infection such as the relative sizes of $\rho$, $\beta$ and $\sigma$.

An important issue in all these partial lockdown scenarios is whether there will be an after-wave once the lockdown policies are lifted. The effective reproduction number at the time lockdowns are lifted tells us whether there will be a second wave or not. Specifically, denoting with $\tau$ the time of lifting the lockdown policies, we have that a second wave will emerge if and only if the effective reproduction number at time $\tau$ is higher than one, i.e. $\gamma(K_{L\tau}, K_{H\tau}, I_\tau)S_\tau\mathscr{R}_0 > 1$.

[25] If the government can enforce the minimal degree of social distancing to every individual, then infectious people will also be forced to exercise the minimal degree of social distancing. In this case, the rates of infection need to be multiplied by $(1-\underline{\delta})$ as only $(1-\underline{\delta})$ proportion of the infectious people may come in contact with susceptible individuals.

### 5 Quantitative analysis

We calibrate the our model to daily UK data on reported deaths from the Covid-19 epidemic, using also existing estimates of epidemiological model parameters that represent the basic reproduction number, the length of the infectious period, and the length of the time from the onset of the infection to death.

Specifically, we set the daily discount rate at $\xi = 0.05/365$ to be consistent with a 5% annual interest rate, and $N = 66.87M$. In the basic calibration, we use the following estimates: $\mathscr{R}_0 = 2.25$, $1/\sigma = 4.5$, $\psi = 17$.[26] We also use $\beta = \mathscr{R}_0\sigma$. We set the introduction date to be $t_0 = \tau_1 - \psi$, where $\tau_1$ is the date in the data when the first death was reported.[27] Furthermore, we postulate that

$$F(w) = \min\{\theta_0^{1-kw}, \overline{p}\}, \qquad \theta_0 \leq \overline{p} \leq 1$$

Observe that this formulation incorporates the special case of a constant mortality rate when $k = 0$. Instead, when $k > 0$, the mortality rate is an increasing and initially convex function of $w$. Under this formulation, a low (but positive) $k$ captures (in a smooth way) a capacity constraint (in critical care) that becomes an issue only for high $w's$, while a high $k$ would capture a capacity constraint that binds early in the epidemic. As we will see below, the calibrated value for $k$ turns out to be large justifying our approach to endogenise the mortality rate of the high-risk individuals in the SIR model with heterogeneity in the infection-induced mortality rates used in Lourenço et al. (2020).

In addition, we set $\overline{\delta} = 0.7$, so that $1 - \overline{\delta}$ is roughly equal to the share of GDP of essential sectors such as health, government, retail, utilities, and food manufacturing, which continue to operate even in extreme scenarios (see also Alvarez et al. 2020). We also set $\underline{\delta} = 0$ to capture an environment without intervention.

We assume logarithmic utility (i.e. CRRA parameter equal to one) and normalise units by setting the flow utility of all susceptible people to be equal to zero, regardless of their risk type. Therefore, the benchmark consumption of all susceptible individuals in our model is normalised to one, and hence all model units are measured in daily

[26] Kucharski et al. (2020) report a median estimate of $\mathscr{R}_0 = 2.35$ and Wu et al. (2020) report an estimate of $\mathscr{R}_0 = 2.2$. Given these estimates, we will also check how our calibration results change when $\mathscr{R}_0 = 2.22$ and $\mathscr{R}_0 = 2.28$. Lourenço et al. (2020) report and use an estimate of $\psi = 17$, stating that this estimate for $\psi$ includes the incubation period as well. Linton et al. (2020), report that the mean time to death from the onset of disease is 15 and the mean incubation period is 5.3. (see their Table 1). Given these estimates, we will also derive calibration results for the cases $\psi = 15$ and $\psi = 20$. Lourenço et al. (2020) also report and use an estimate of $1/\sigma = 4.5$. We will also conduct sensitivity analysis by using $\sigma = 0.18$ and $\sigma = 0.205$.

[27] To be more precise, the introduction date thus found is 18/2/2020. There were only 9 reported cases during the 18 days *prior* to the introduction date and no newly reported cases on the introduction date. From these 9 reported cases, the first 2 were on 31/1/2020, the next one on 7/2/2020, with 5 more on 10/2. The last reported case prior to the introduction date was on 12/2/2020 and no other case was reported until 24/2/2020. Of course, most probably not all cases were reported as symptoms can be similar to flu, which motivated our focus on deaths for the calibration of our model. Moreover, in reality there is heterogeneity in the time of delay from the onset of illness to death and in the incubation period. In fact, Linton et al. (2020), find that the time of delay from the onset of illness to death follows a lognormal distribution with mean 15 and standard deviation 6.9, while the incubation period follows a lognormal distribution with mean 5.3 and standard deviation 3.2, Taking into account of such heterogeneity is left for future work.

**Table 1** Calibration: Exogenous parameters

| Definition | Symbol | Value |
|---|---|---|
| CRRA parameter | – | 1 |
| Recovery rate | $\sigma$ | 1/4.5 |
| Infection rate | $\beta$ | 0.5 |
| Mean length between onset and death | $\psi$ | 17 |
| Annual interest rate | $r$ | 5% |
| Daily discount rate | $\xi$ | $r/365$ |
| Population | $N$ | $66.87M$ |
| Percentage of Essential Sectors | $(1-\overline{\delta}) \times 100$ | 30% |
| Economic cost of death | $\overline{K}$ | 108 |

consumption per capita terms. We also set $\overline{K} = 108$ which is consistent with a statistical value of life of 127.5 times the annual consumption per capita. Table 1 summarises the set parameters. To see how a statistical value of life of 127.5 times the annual consumption per capita implies $\overline{K} = 108$, let, first, $P$ be the extra cost of dying in terms of current annual consumption per capita. We identify a statistical value of life SVL with the shadow cost of dying (in the absence of an epidemic), which consists of the NPV of one unit of annual per capita consumption (with $r$ annual discount rate) plus the extra cost of dying $P$, i.e. equal to $\frac{1}{r} + P$. With $SVL = 127.5$ and $r = 0.05$, we have $P = 107.5$. Next, note that a cost $P$ is equivalent to paying daily a constant stream of $\xi P$. Given that $\overline{K}$ is the NPV of the extra cost of dying (in $\psi$ periods) in utility terms, and utility is logarithmic, we can think of $\overline{K}$ as being equal to $\overline{K} = \frac{log(1)}{\xi} - \frac{log(1-\xi P)}{\xi}$. Using $P = 107.5$ and $\xi = 0.05/365$ gives $\overline{K} = 108$. Observe now that a statistical value of life of 127.5 times the annual consumption per capita is equal to the average of the value of 30 times the annual consumption per capita found by Hall et al. (2020) and used in Alvarez et al. (2020) and the value of 225 times the annual consumption per capita found by Kniesner and Viscusi (2020).[28]

Finally, we calibrate the remaining parameters by minimising the squared sum of the residuals (expressed in proportional terms) between cumulative deaths in our model, implied by (12), and those in UK data.[29]

[28] Farboodi et al. (2020) uses, in terms of our notation, $\xi P = 0.011$ and thereby $\overline{K} = 80$. Estimates of the statistical value of life are typically attained under the assumptions of no epidemic and a constant mortality rate. In our model, however, we have an endogenous probability of dying (after $\psi$ periods) which depends on the level of infection. Moreover, the flow utility (in consumption-equivalent units) of infected and recovered individuals are typically (weakly) lower than that of susceptible individuals. We assume here that the duration of the epidemic is small enough, so that the implied cost of death from existing estimates of the statistical value of life remains a good approximation of the extra cost of death in our model. Note, however, that we will also perform sensitivity analysis using lower as well as higher values for the cost of death within the range [10, 208] implied by the range of statistical values of life [30, 225] and an annual discount rate 5%.

[29] A time series was obtained from the John Hopkins University Centre for Systems Science and Engineering COVID-19 GitHub repository. Available: at https://github.com/CSSEGISandData/COVID-19. Following Lourenço et al. (2020), we trimmed the data to the first fourteen days of death counts above

Given the integer nature of our data, matching all data points is futile especially when there is a significant jump in values (in proportionate terms). To deal with this, we weight the residuals (in proportional terms) between our model-generated counts of cumulative deaths and the actual data by using the following procedure. First, we attempt to calibrate the model by assigning weight one on each residual. This typically produces results with large residuals for the fifth and eighth data points. The reason is that both data points are associated with an increase in the number of cumulative deaths which is more than 200%, something that the model cannot match. In fact, in attempting to reduce these large residuals, the model was not fitting well the other moments as well. Motivated by this, in the second step we assign weight zero on the two largest residuals. Calibration with this weight profile typically produces larger residuals for the two ”ignored” data points than the uniform weight rule but much better approximation of the other data points.

#### 5.1 The equilibrium SIR case

The remaining to be calibrated parameters are $\rho, \theta_0, k, \overline{p}$, $\phi_L, \phi_H$, $K_{L,0}$, $K_{H,0}$ and $I_0$. We find $\rho = 0.1828$, $\theta_0 = 0.1974$, $k = 1091332$, $\overline{p} = 0.4333$, $I_0 = 38/N$, $\phi_L = -0.0025$, $\phi_H = -2.9925$, $K_{L,0} = 5.58$, $K_{H,0} = 14513$. Observe that $0 < \rho < 1$, and so the data on UK deaths suggest that the postulated heterogeneity is empirically plausible. In the next subsection, we discuss the benchmark case of no heterogeneity to determine the bias from calibrating such a model by using data that is generated by a heterogenous population.

Figure 2 shows reported and model generated counts of cumulative deaths ($log10$ transformed). Ignoring the residuals for the two dates when the proportional increase in deaths was more than 200%, we have that the absolute residuals in proportional terms are below 6.2% for the first four and last six dates. For the remaining two dates we have that the model predicts only one count of cumulative deaths more than the actual death toll of nine deaths on 12/03/2020, and predicts only one count of cumulative deaths less than the actual death toll of seven on 11/03/2020.

This model predicts a very high death toll of $1,406,630$ deaths in the absence of a government intervention. It also predicts that by the end of the epidemic 60.9% of the UK population would have been exposed to the virus, and that infections reach their peak of 7.58% of the UK population by the 19th of April. Moreover, this model predicts that by the date of the first reported death 0.009% would have already been infected with the virus. This last number is noticeably lower than the prediction of 0.08% in Lourenço et al. (2020). Interestingly, our model also predicts that in the absence of intervention the current epidemic wave in the UK would last for around six months and a mean case fatality rate of around 7.84% over the duration of the epidemic.

Footnote 29 continued
zero until 19/03/2020 (06/03/2020 to 19/03/2020) to include only the initial increase free of effects from local control measures to avoid contamination by mitigation strategies that were implemented after that time. The data was accessed on 04/04/2020 the first time. UK data was updated on 31/04/2020 and was accessed on 01/05/2020. This version of the paper uses the updated data.

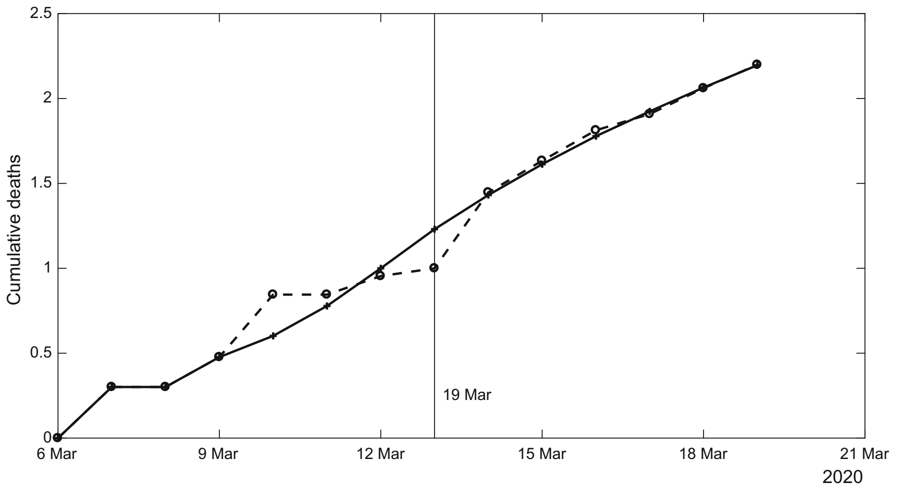

**Fig. 2** The model fit. The dash line shows the actual data, while the solid line depicts the model data

The equilibrium model generates also endogenous social distancing on the part of susceptible individuals. In the calibrated model, the endogenous mean contact rate, when averaged over the duration of the epidemic, is 0.8138. In the next subsection, we discuss the benchmark case of exogenous and constant contact rate to determine the bias from calibrating such a model by using data that is generated under endogenous social distancing.

As Fig. 3 shows, high-risk individuals exercise maximal social distancing around the infection peak, and certainly before the announcement of the UK lockdown on the evening of the 23rd of March. High-risk individuals exercise a much stricter social distancing than low-risk individuals, reaching maximal social distancing before the infection peaks and maintaining it after the infection peaks. Social distancing of low-risk individuals is increasing with the level of infection, reaches a peak and then decreases with the level of infection. The mean contact rate has a U-shape for most part of the epidemic emphasising further the importance of incorporating endogenous social distancing in the SIR model (which assumes that the mean contact rate is constant throughout the epidemic).[30]

Figure 4 contrasts social distancing by the various risk types against one of its determinants; namely, the level of infections. Interestingly, the peak of low-risk individuals' social distancing is after the peak of infection. To understand this, note that, as we have already mentioned when discussing the equilibrium, social distancing is

[30] The calibration results are very similar when we assume that the mortality rate is given by the logistic function $F(w) = \frac{\overline{p}}{1+e^{-k(w-x)}}$: $x = 9.4/10^8$, $k = 1.1976 \times 10^7$, $\rho = 0.1527$, $\overline{p} = 0.5239$ and $I_0 = 37/N$, and $\phi_L = -0.0028$, $\phi_H = -2.9175$, $K_{L,0} = 6.4035$, $K_{H,0} = 14171$. Moreover, the peak date is on the 17th of April, the peak level of infection is 7.35%, the proportion in the population of infected by the date the first death was reported is 0.009%, the duration of the epidemic is about 6 months, the level of exposure at the end of the epidemic is 60.62%, and the total number of deaths is around $1.4M$. Finally, the mean case fatality rate is around 7.93% over the duration of the epidemic, and the mean contact rate, when averaged over the duration of the epidemic, is 0.8208.

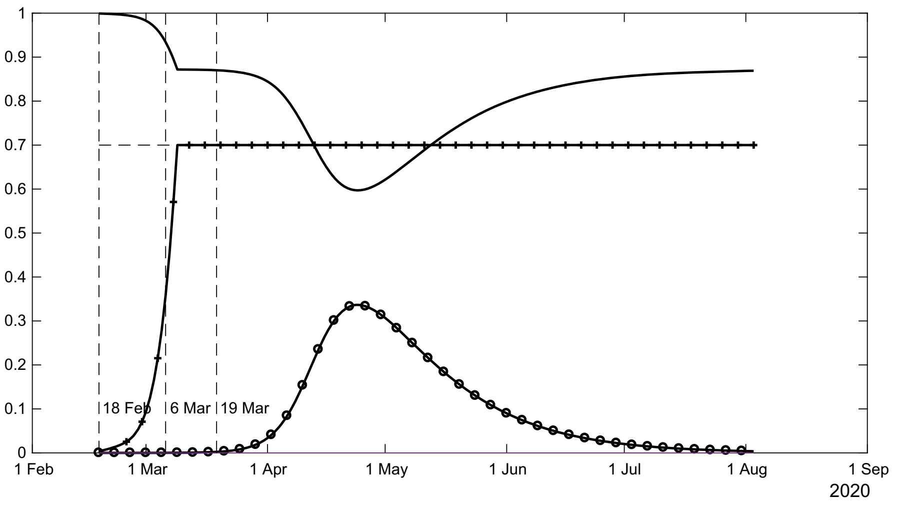

**Fig. 3** Equilibrium social distancing and the mean contact rate. The vertical dash lines indicate the dates 18 February, 6 March and 19 March. The horizontal dash line indicates the maximal level of social distancing. The plain solid line depicts the mean contact rate, while the circle line shows the social distancing of low-risk individuals and the cross line the social distancing of high-risk people

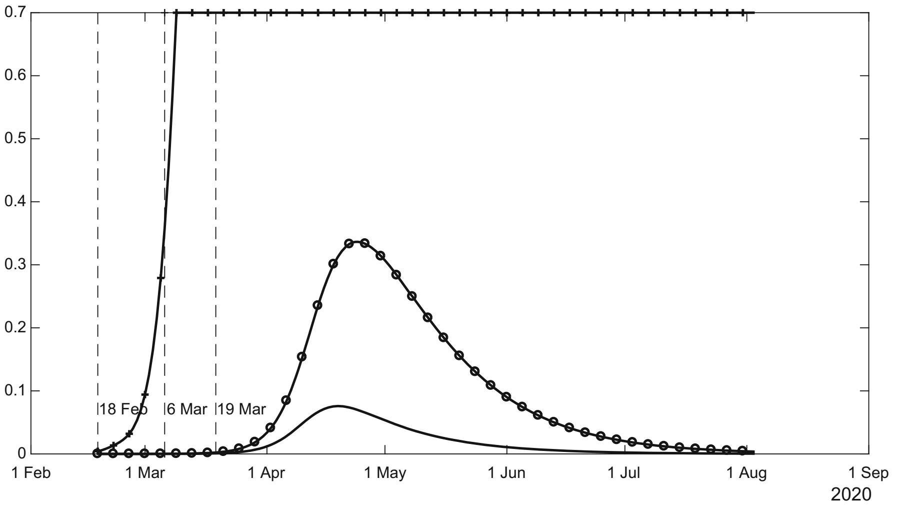

**Fig. 4** Equilibrium social distancing against infections. The vertical dash lines indicate the dates 18 February, 6 March and 19 March. The plain solid line depicts infections as a percentage of the UK population, while the circle line shows the social distancing of low-risk individuals and the cross line the social distancing of high-risk people

(weakly) increasing with the level of infection while keeping constant the expected NPV of the welfare cost from getting infected. Moreover, social distancing is increasing with the expected NPV of the welfare cost from getting infected while keeping constant the level of infection. However, the expected NPV of the welfare cost from getting infected and the level of infection may not move in sync during the epidemic. To see why this is the case, recall (13). Using the envelope theorem, we can see that an increase in infections leads to an increase in the cost from getting infected in the next instance. Observe, however, that how this cost evolves over time depends also on its current value and the flow utilities from being in the Infected and Recovered states, as captured by the term $\phi_L + \xi K_L$. This term can be positive for high levels of $K_L$. In fact, Fig. 4 highlights that in the calibrated model this is indeed the case at the infection peak, making low-risk individuals' social distancing being increasing on the date of the infection peak and for some time after.

Next, we check how the predictions about the epidemic and social distancing (captured by the equilibrium mean contact rate) change when we consider alternative values for $\overline{K}$, $\mathscr{R}_0$ (equivalently, $\beta$), $\sigma$ and $\psi$. For all the reported alternative values, the duration of the current UK epidemic would have been around 6 months in the absence of government intervention. Moreover, the mean fatality rate is around 0.795. Different times from the onset of infection to death – for instance, $\psi = 15, 20$ – lead to similar predictions for the fraction in the population of high-risk individuals, the peak level of infection, the death toll and total exposure to the virus as well as the initial level of infection and the number of cases when the first death was reported. The only difference is in the peak date, with $\psi = 15$ predicting 22nd of April, and $\psi = 20$ predicting 16th of April.[31] Table 2 reports the findings for alternative values for $\overline{K}$, $\mathscr{R}_0$ and $\sigma$ (parameters that are not stated in the heading of a column stay the same as in the basic calibration).

We note the reduction in the infection peak, the total exposure at the end of the epidemic and the death toll as the transition rate from the Infectious to Recovered state increases. The reason is that faced with a lower duration of the infection and thereby lower infectiousness of the virus (recall that $\mathscr{R}_0$ decreases as $\sigma$ increases), low-risk individuals respond with lower social distancing. The latter, in turn, makes the high-risk individuals to increase significantly their social distancing (at the early stages of the epidemic), which leads to an increase in the mean contact rate when averaged over time.

Observe next that as the economic cost of death increases, the death toll, total exposure, the infection peak and the mean contact rate, when averaged over the duration of the epidemic, have a U-shape. The reason behind these non-monotonicities is that as the economic cost of death increases high-risk individuals take relatively more precautions at the early stages of the infection, which, in turn, reduces (at the next unit of time) the risk of infection and thereby the willingness of low-risk individuals to exercise social distancing. The relative strength of these two opposite effects on social distancing influences the net impact of a variation in the economic cost of death on the various dimensions of the epidemic. In fact, as the reported results indicate, the net impact depends on the level of the economic cost of death.

31 Details are available upon request.

**Table 2** Sensitivity analysis

| | Main Calibration<br>$\sigma = 0.222$; $\beta = 0.5$; $\overline{K} = 108$ | $\sigma = 0.205$<br>($\mathscr{R}_0 = 2.44$) | $\sigma = 0.18$<br>($\mathscr{R}_0 = 2.78$) | $\mathscr{R}_0 = 2.28$<br>($\beta = 0.507$) | $\mathscr{R}_0 = 2.22$<br>($\beta = 0.493$) | $\overline{K} = 130$ | $\overline{K} = 80$ |
|---|---|---|---|---|---|---|---|
| Mean contact rate (avg) | 0.8138 | 0.7895 | 0.7413 | 0.847 | 0.8667 | 0.856 | 0.863 |
| Infection peak | 7.58% | 9.44% | 11.11% | 8.51% | 9.25% | 9.14% | 9.26% |
| Peak date | 19 April | 20 April | 19 April | 15 April | 16 April | 16 April | 16 April |
| Death toll | 1.4$M$ | 1.56$M$ | 1.78$M$ | 1.46$M$ | 1.43$M$ | 1.44$M$ | 1.45$M$ |
| Exposure | 60.9% | 65.17% | 68.23% | 63.65% | 65.2% | 64.92% | 65.24% |
| Initial infection pool | 38 | 32 | 28 | 33 | 39 | 35 | 35 |
| Infections by 6 March | 0.009% | 0.0088% | 0.01% | 0.0086% | 0.0087% | 0.0084% | 0.0085% |

Note finally that an increase in the basic reproduction number implies a higher transmission rate but, also, higher social distancing as an increase in infectiousness leads to a higher probability of getting infected. The net result on the various dimensions of the epidemic will depend on the relative strength of these two effects. The reported results in Table 2 suggest that the death toll, the infection peak, total exposure and mean contact rate, when averaged over the duration of the epidemic, have a U-shape as the basic reproduction number increases.[32]

### 5.2 Comparison with some benchmark cases

#### 5.2.1 Exogenous social distancing

We start with the case of the epidemiological version of the SIR where social distancing is treated as exogenous and constant. In this benchmark case, we define the exogenous mean contact rate to be $\gamma = 1-\delta$. This benchmark model is, in effect, a generalisation of the model in Lourenço et al. (2020), who assume that the mortality rate is exogenous and that there is zero social distancing by every individual. Here, however, we allow for $\delta$ to be positive. In effect, under the assumption that all individuals exert the same level of social distancing, the proportion in the population of the high-risk individuals in Lourenço et al. (2020) is equal (in terms of our notation) to $\rho(1-\delta)$. Importantly, here, $\delta$ is also calibrated from the data together with the rest of the model parameters.[33] We find $\delta = 0.0913$, $\rho = 0.142$, $\theta_0 = 0.09$, $k = 636016$, $\overline{p} = 0.5632$ and $I_0 = 78/N$. Interestingly, $\rho(1 - \delta) = 0.129$, which is significantly higher than the proportion in the population of the high-risk individuals found in Lourenço et al. (2020) under the assumption of exogenous mortality rate. Observe also that the mean contact rate in this benchmark model, $1-\delta$, is higher by about 10% than the mean contact rate, when averaged over the duration of the epidemic, under endogenous social distancing.

As our model, this benchmark model predicts that the current epidemic wave in the UK would last for around six months in the absence of intervention. However, it also predicts a total number of deaths of about $4,33M$ and infections reaching their peak of 16.11% of the UK population by 17th of April. It also predicts that by the end of the epidemic 80.86% of the UK population would have been exposed to the virus. Our model with endogenous social distancing predicts one third less, about half less, two days later, and one quarter less of these findings, respectively. Thus, the model version with constant mean contact rate significantly over-predicts the death toll, exposure and the infection peak. Moreover, this benchmark model predicts a mean case fatality rate of 6.78% which is about one percent below the one predicted by the calibrated model with endogenous social distancing. In addition, this benchmark model predicts that by the date of the first reported death 0.011% would have already been infected with the virus. The latter is around 86% lower than the number found in Lourenço et al.

[32] For completeness, we also note here that the calibrated fraction in the population of the high-risk individuals, starting from the left column, is 0.275, 0.302, 0.063, 0.109, 0.123, 0.108.

[33] Ignoring the residuals for the two dates when the proportional increase in deaths was more than 200%, we have that the absolute residuals in proportional terms are below 5% for the first four, the sixth and the last three dates. For the remaining four dates the residuals in proportionate terms are below 14%.

(2020), and 22% higher than the number we found above under endogenous social distancing.

##### 5.2.2 No heterogeneity in mortality rates

In this section we want to compare our main calibration results with those under the alternative assumption that there is no heterogeneity in mortality rates. We should emphasise here that, as we have seen above, the calibrated value of $\rho$ when we allow it to take any value between zero and one is indeed positive but less than one justifying our focus on heterogeneity in terms of underlying health conditions and how they may lead to a death from infection.

Given that in the data there are deaths, to give the best chance to the benchmark model with no heterogeneity, we assume that in this model all individuals face a risk of developing a serious disease from infection and die after $\psi$ periods, i.e. $\rho = 1$. This benchmark model is, in effect, a generalisation of Toxvaerd (2020) allowing also for (endogenous) mortality.[34]

We find $\theta_0 = 0.0336$, $k = 98139$, $\overline{p} = 0.8$ and $I_0 = 38/N$. This benchmark model predicts a mean case fatality rate of 7.88% which is very similar to the one predicted by the calibrated model with heterogeneity. However, this benchmark model massively under-predicts, compared to the model with heterogeneity, the death toll, the infection peak and total exposure in the absence of a government intervention, predicting a total number of deaths of about 12,000, that infections reach their peak of 0.0075% of the UK population by 14th of May, and that by the end of the epidemic only 0.25% of the UK population would have been exposed to the virus. In addition, this benchmark model predicts that by the date of the first reported death 0.0065% would have already been infected with the virus. The latter is around 28% lower than the number we found earlier under heterogeneity. Finally, in this model, the endogenous mean contact rate, when averaged over the duration of the epidemic, is 0.5056 which is around 38% lower than the corresponding mean contact rate under heterogeneity. The reason for these discrepancies is that in an homogenous population where everyone is at risk of dying from infection, everyone exercises a high degree of social distancing. Interestingly, however, no one exercises the maximal degree of social distancing. The reason is that when everyone else is expected to exercise high social distancing the chances of getting infected are low and so it is not optimal to exercise maximal social distancing.

Table 3 summarises the comparison between our model and the two benchmark models discussed above.

#### 5.3 Partial lockdowns in the equilibrium SIR model

In what follows, we examine the effects on the main predictions of our model of various scenarios of government intervention regarding social distancing. To fix ideas, we assume that the controlling measures are implemented on the 24th of March, i.e. the first day of the UK lockdown as announced by the UK government at 8pm of

[34] In Toxvaerd (2020) there is no mortality. To compare our model with the one there we would have to set $\rho = 0$, which would however predict zero deaths throughout the epidemic.

**Table 3** Comparison of models

| | Our model | Exog. social distancing | No heterogeneity ($\rho = 1$) |
|---|---|---|---|
| Mean contact rate (avg) | 0.8138 | 0.9087 | 0.5056 |
| Infection peak | 7.58% | 16.11% | 0.0075% |
| Peak date | 19 April | 17 April | 14 May |
| Death toll | $1.4M$ | $4.33M$ | 12,000 |
| Exposure | 60.9% | 80.86% | 0.25% |
| Infections by 6 March | 0.009% | 0.011% | 0.0065% |

the 23rd of March, and are lifted on a specific date at least 5 weeks after the 24th of March. We examine the case where both dates were not known on the introduction date. We also assume that, on the date the lockdown was announced, the lockdown was unanticipated, whereas the date the lockdown is lifted was anticipated.[35]

First, we run the case where the government enforces a minimal level of social distancing whereby *all* individuals cut at least 40% of their contacts until the 30th of April (inclusive). Under such regime, low-risk susceptible individuals are forced to choose $\delta_L^* = 0.4$ throughout the intervention period, while the behaviour of high-risk susceptible individuals is largely unaffected.[36] All infectious individuals are also forced to cut 40% of their contacts. In this scenario, the mean contact rate is, with some abuse of notation, equal to $\gamma(1 - 0.4)$. Figure 5 shows the social distancing of susceptible individuals depending on their risk type under this partial lockdown policy. Similar patterns of social distancing are found for longer periods of such social distancing restrictions.

We find that under such an intervention the death toll is reduced by around 10,000, about 0.8% reduction compared to the death toll under no intervention. We also find that under such an intervention 60.71% of the UK population would have been exposed to the virus by the end of the epidemic, which is lower only by 0.19% than the total exposure by the end of the epidemic without intervention. Finally, infections would have reached their peak of 7.33% by the 6th of June, which are lower by 0.25% and almost a month and a half later, respectively, compared to the no intervention case. Enforcing minimal level of social distancing at $\underline{\delta} = 0.5$ would have a larger impact. Namely, compared to the case of no intervention, the death toll would have been reduced by about 1.07%, the total exposure would have been lower by 0.32%, while the infection peak would have been lower by around 0.2% and reached by around two months later.

[35] In reality, the situation is somewhere between with individuals having (some) imperfect information on either of the two dates. We leave it for future work to appropriately modify our model to incorporate imperfect information on the part of individuals regarding government intervention on social distancing.

[36] The highest degree of social distancing low-risk susceptible individuals would choose in the absence of government intervention would be 0.336. We have also run the case when the government enforces a minimal level of social distancing whereby individuals cut at least 34% of their contacts until the 30th of April (inclusive). Under such regime, infectious and low-risk susceptible individuals are forced to choose $\delta_L^* = 0.34$ throughout the intervention period. We find that such an intervention would only have a small effect in the infection peak, level of exposure and death toll. It would also delay the infection peak by a month.

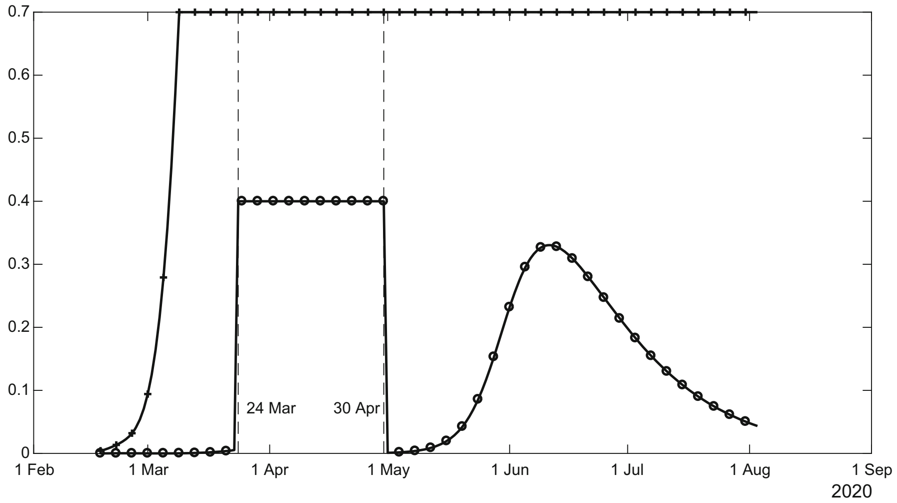

**Fig. 5** Equilibrium social distancing under minimal social distancing of $\underline{\delta} = 0.4$ until 30 April. The vertical dash lines indicate the dates 24 March and 30 April. The circle line shows the social distancing of low-risk individuals and the cross line the social distancing of high-risk people

Extending the lockdown of non-essential sectors and thereby the minimal level of social distancing after the 30th of April would increase the impact of this type of lockdown policies. For instance, keeping the minimal level of social distancing at $\underline{\delta} = 0.4$ until the 30th of May (inclusive) would have as an effect that the death toll would have been reduced by about 1.2%, the total exposure would have been lower by around 0.35%, and the infection peak would have been lower by 0.29% and reached by about three months later compared to the case of no intervention. All aforementioned interventions reduce the mean contact rate, when averaged over time, by around half. Figures 6, 7 and 8 contrast the infections, exposure and the death toll under no intervention, and under minimal social distancing of $\underline{\delta} = 0.4$ until the 30th of April and until the 30th of May. Figures 5 and 6 together highlight the impact the social distancing decisions of the low-risk susceptible individuals have on the evolution of the epidemic: as soon as the lockdown measures in question are lifted there is an after-wave due to low-risk susceptible individuals increasing drastically the frequency of their interactions.

In our model individuals are assigned utilities and therefore we can measure the welfare impact of the interventions in question. Assuming that the flow utilities of the high- and low-risk susceptible individuals and the flow utilities of the high- and low-risk recovered individuals coincide, we find that forcing all individuals to reduce their contact rate to 0.4 until the 30th of April would yield an increase in the current value of total lifetime utility from the time of the intervention onwards equal to 0.6% of the corresponding current value of total lifetime utility under no intervention. The corresponding welfare gains from forcing all individuals to reduce their contact rate to 0.4 until the 30th of May and by 0.5 until the 30th of April are equal to 1.11% and 0.83%, respectively. Interestingly, these findings suggest that milder but longer

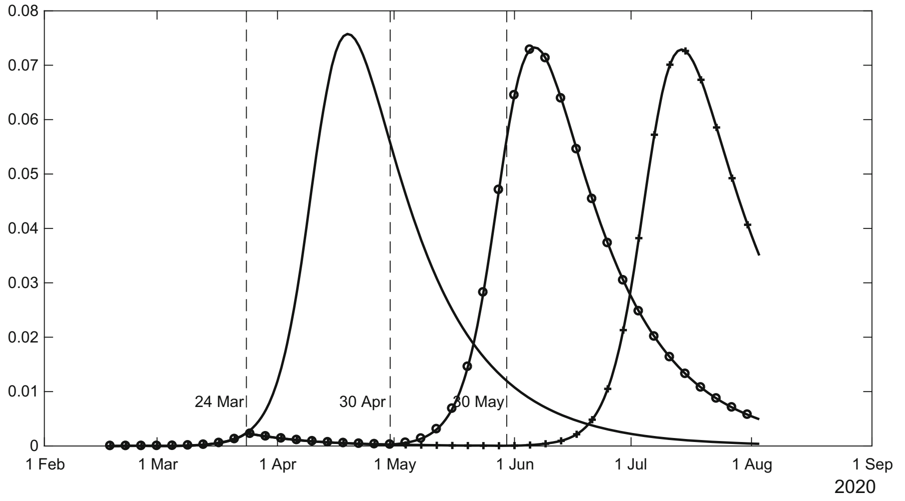

**Fig. 6** Infections (as a percentage of the UK population) under no intervention and minimal social distancing of $\underline{\delta} = 0.4$ until 30 April or 30 May. The vertical dash lines indicate the dates 24 March, 30 April and 30 May. The plain solid line depicts infections in the absence of any intervention, while the circle line shows infections under minimal social distancing of $\underline{\delta} = 0.4$ until 30 April and the cross line infections under minimal social distancing of $\underline{\delta} = 0.4$ until 30 May

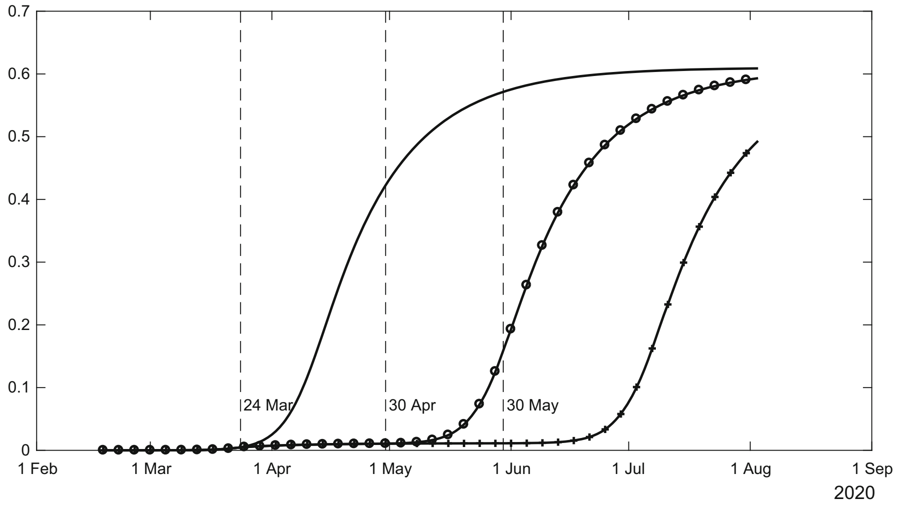

**Fig. 7** Exposure (as a percentage of the UK population) under no intervention and minimal social distancing of $\underline{\delta} = 0.4$ until 30 April or 30 May. The vertical dash lines indicate the dates 24 March, 30 April and 30 May. The plain solid line depicts infections in the absence of any intervention, while the circle line shows infections under minimal social distancing of $\underline{\delta} = 0.4$ until 30 April and the cross line infections under minimal social distancing of $\underline{\delta} = 0.4$ until 30 May

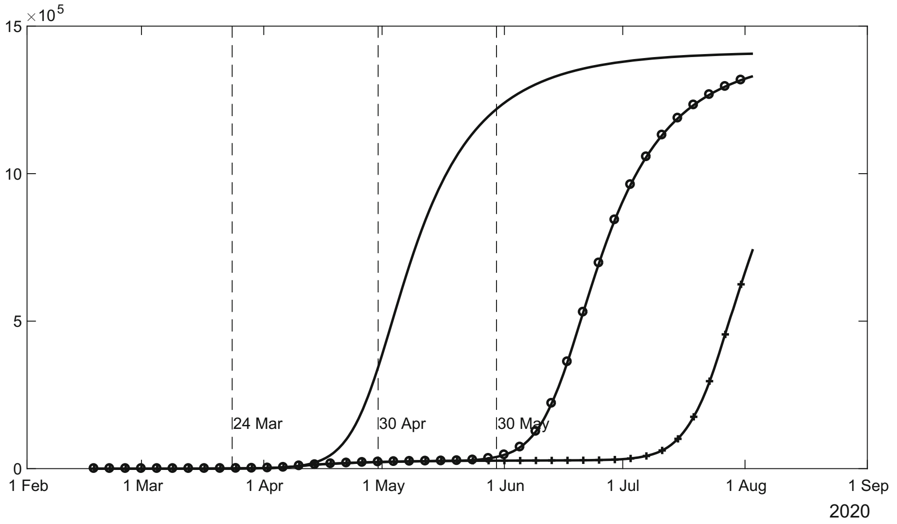

**Fig. 8** Cumulative deaths (as a percentage of the UK population) under no intervention and minimal social distancing of $\underline{\delta} = 0.4$ until 30 April or 30 May. The vertical dash lines indicate the dates 24 March, 30 April and 30 May. The plain solid line depicts infections in the absence of any intervention, while the circle line shows infections under minimal social distancing of $\underline{\delta} = 0.4$ until 30 April and the cross line infections under minimal social distancing of $\underline{\delta} = 0.4$ until 30 May

**Table 4** Impact of increasing minimal degree of social distancing $\underline{\delta}$

| | No interv. | $\underline{\delta} = 0.4$ til 30 April | $\underline{\delta} = 0.4$ til 30 May | $\underline{\delta} = 0.5$ til 30 April |
|---|---|---|---|---|
| Mean contact rate (avg.) | 0.8138 | 0.4588 | 0.4436 | 0.3755 |
| Infection peak | 7.58% | 7.33% | 7.29% | 7.38% |
| Peak date | 19 April | 6 June | 14 July | 14 June |
| Death toll | 1.4$M$ | 1.39$M$ | 1.39$M$ | 1.39$M$ |
| Total exposure | 60.9% | 60.71% | 60.55% | 60.58% |
| Welfare gain | – | 0.6% | 1.11% | 0.83% |

interventions of this type might be better in terms of total welfare. The impact of increasing the minimal degree of social distancing $\underline{\delta}$ is summarised in Table 4.

Next, we derive the epidemic under several alternatives values for the maximal level of social distancing, $\overline{\delta} = 0.70, 0.71, 0.72, \ldots, 0.99, 1$ until the 30th of April (inclusive). This type of intervention does not affect the behaviour of infectious individuals. Under all these cases the behaviour of the low-risk susceptible individuals remains largely unchanged.[37] Since they are the vast majority of susceptible individuals in the calibrated economy, it thus should not be a surprise that we find that such intervention

[37] To be precise their social distancing increases somewhat once the intervention stops because the risk of getting infected is somewhat lower at that point due to the high-risk individuals having being more cautious during the intervention period.

has a very small impact on the pools of infectious and susceptible individuals.[38] A consequence of this is that in the calibrated economy there is no after-wave when these lockdown policies are lifted, simply because the endogenous mean contact rate is small enough so that the effective reproduction number is less than one when these lockdown policies are lifted.

However, such an intervention enables the high-risk susceptible individuals to increase their social distancing during the intervention period up to the new maximal level; Fig. 9 depicts social distancing by susceptible individuals when $\overline{\delta} = 0.8$. Therefore, such an intervention has a significant impact on the death toll of the current UK epidemic. When the maximal social distancing increases to $\overline{\delta} = 0.8$ until the 30th of April, the death toll is reduced by around 300,000 ( 21%) compared to the case of no government intervention. As another case, consider when maximal social distancing increases to $\overline{\delta} = 0.9$ until the 30th of April. In this case, the death toll is reduced by around 600,000 (43%) compared to the case of no government intervention.

Furthermore, interventions of this type that last between 5 and 8 weeks have very similar effects on the pools of infectious and susceptible individuals. The reason here is that high-risk susceptible individuals already exercise the maximal degree of social distancing before the start of the lockdown period and maintain maximal social distancing well beyond the end of an 8-week lockdown period. However, interventions that last between 5 and 8 weeks do differ in terms of the size of the negative effect they have on the death toll. For instance, increasing maximal social distancing to $\overline{\delta} = 0.8$ until the 30th of May would reduce the death toll by 430,000 (around 30.7%) compared to the case of no government intervention. All aforementioned interventions lead to very similar mean contact rates, when averaged over time. Figure 10 depicts the death toll when the maximal social distancing increases to $\overline{\delta} = 0.8$ until the 30th of April and until the 30th of May.

Assuming that the flow utilities of the high- and low-risk susceptible individuals and the flow utilities of the high- and low-risk recovered individuals coincide, we find that allowing high-risk individuals to reduce their contact rate to 0.8 until the 30th of April would yield an increase in the current value of total lifetime utility from the time of the intervention onwards equal to 16% of the corresponding current value of total lifetime utility under no intervention. The corresponding welfare gains from allowing high-risk individuals to reduce their contact rate to 0.8 until the 30th of May and to 0.9 until the 30th of April are equal to 26% and 31%, respectively. Interestingly, these findings suggest that allowing for stricter restrictions on essential activities but for a shorter period might be better in terms of welfare gains. This is in contrast to what the results in Table 4 indicate regarding the welfare effects of restricting less essential activities. The impact of increasing the maximal degree of social distancing $\overline{\delta}$ is summarised in Table 5. Comparing the welfare effects in Tables 4 and 5 we also observe that allowing the operation of only 20% the more essential sectors (i.e. increasing maximal social distancing from 0.7 to 0.8) can generate much larger welfare gains than shutting down

[38] For instance, for the cases of $\overline{\delta} = 0.7, 0.8, 0.9$ until the 30th of April, the peak infection is 7.59%, 7.23%, 6.87%, respectively, the dates of the peak are 19th, 20th and 21st of April, respectively, and the proportion in the population of the exposed individuals at the end of the epidemic is 61%, 60.8%, 60.62%, respectively.

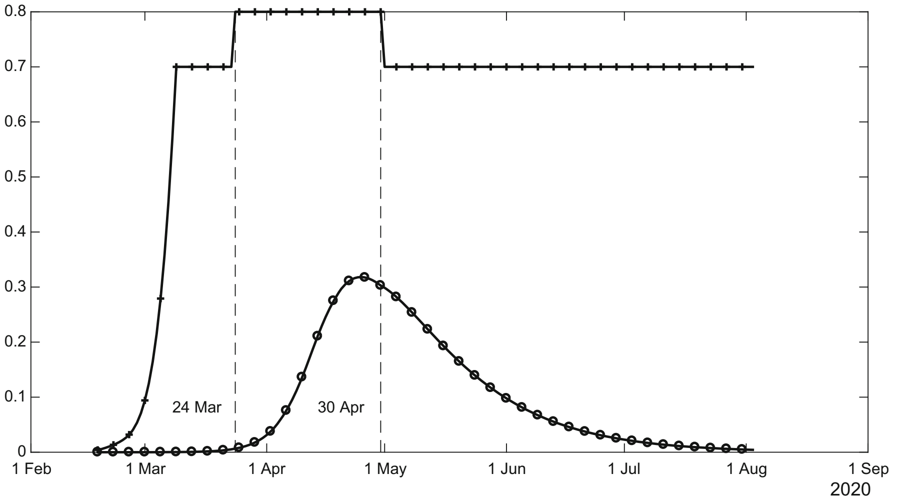

**Fig. 9** Equilibrium social distancing under maximal social distancing of $\underline{\delta} = 0.8$ until 30 April. The vertical dash lines indicate the dates 24 March and 30 April. The circle line shows the social distancing of low-risk individuals, while the cross line shows the social distancing of high-risk people

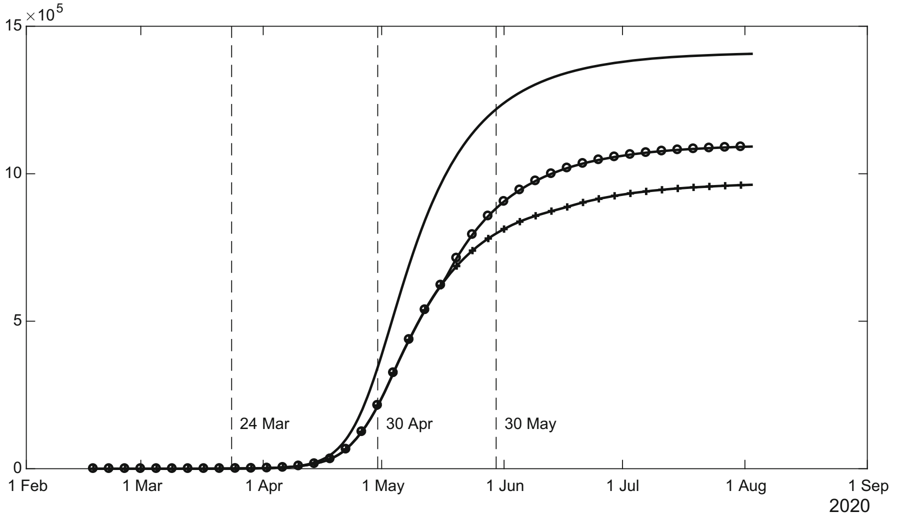

**Fig. 10** Death toll (as a percentage of the UK population) under no intervention and maximal social distancing of $\underline{\delta} = 0.8$ until 30 April and until 30 May. The vertical dash lines indicate the dates 24 March, 30 April or 30 May. The plain solid line depicts deaths in the absence of any intervention, while the circle line shows deaths under maximal social distancing of $\overline{\delta} = 0.8$ until 30 April and the cross line deaths under maximal social distancing of $\overline{\delta} = 0.7$ until 30 May

40% of the less essential sectors (i.e. increasing minimal social distancing from 0 to 0.4).

Our model can match observed numbers of death to a good degree with the appropriate choice of minimal and maximal social distancing. At the time of writing these

**Table 5** Impact of increasing maximal degree of social distancing $\overline{\delta}$

| | No interv. | $\overline{\delta} = 0.8$ til 30 April | $\overline{\delta} = 0.8$ til 30 May | $\overline{\delta} = 0.9$ til 30 April |
|---|---|---|---|---|
| Mean contact rate (avg.) | 0.8138 | 0.8213 | 0.8307 | 0.8182 |
| Infection peak | 7.58% | 7.23% | 7.19% | 6.87% |
| Peak date | 19 April | 20 April | 20 April | 21 April |
| Death toll | $1.4M$ | $1.1M$ | $0.97M$ | $0.81M$ |
| Exposure | 60.9% | 60.8% | 60.18% | 60.62% |
| Welfare gain | – | 16% | 26% | 31% |

lines (7 May 2020) the number of deaths in UK was between 32,000 and 33,000, with the UK (partial) lockdown still being in place, and talks in the news about lifting some of the lockdown measures start appearing in mainstream newspapers.[39] We have checked some indicative levels of $\underline{\delta}$ and $\overline{\delta}$ in an attempt to approximate the aforementioned death toll. It turns out that setting $\underline{\delta} = 0.315$ and $\overline{\delta} = 0.75$ between 24 March and 30 May produces a death toll on the 7th of May between 32,000 and 33,000. Therefore, our model could predict the observed data to a good degree.[40] With such lockdown policies, infectious individuals cut down their contacts by 31.5%. Moreover, the pattern of social distancing by susceptible individuals is similar to the one depicted in Fig. 5 because the low-risk individuals are a significant majority of susceptible people in the calibrated population. Such an intervention leads to a mean contact rate, when averaged over time, of 0.5171, and yields an increase in the current value of total lifetime utility from the time of the intervention onwards equal to 1.23% of the corresponding current value of total lifetime utility under no intervention. Figures 11, 12 and 13 depict the evolution of infections, total exposure and death toll under the lockdown policy that enforces $\underline{\delta} = 0.315$ and $\overline{\delta} = 0.75$ until 30th of May, contrasting them with their counterparts under no intervention.

### 6 Conclusions

Motivated by the current coronavirus epidemic, we have analysed a SIR model of an epidemic but with endogenous social distancing. We have calibrated the model to UK data on reported deaths prior to the introduction of the UK lockdown to study various hypothetical scenarios of government intervention regarding social distancing. We have explicitly taken into account that there is heterogeneity among the population in terms of infection-induced fatality rates and thereby private decisions on social distancing. We have also postulated that, due to limited resources available for health services, mortality rates may depend on the stock of infected people who become seriously ill because of the infection.

[39] See, for instance, the following article in the Independent: https://www.independent.co.uk/news/uk/politics/coronavirus-uk-lockdown-measures-lift-announcement-boris-johnson-news-a9501441.html.

[40] Increasing the maximal degree of social distancing over 75% would enable matching the observed death toll by using a lower minimal degree of social distancing than 31.5%.

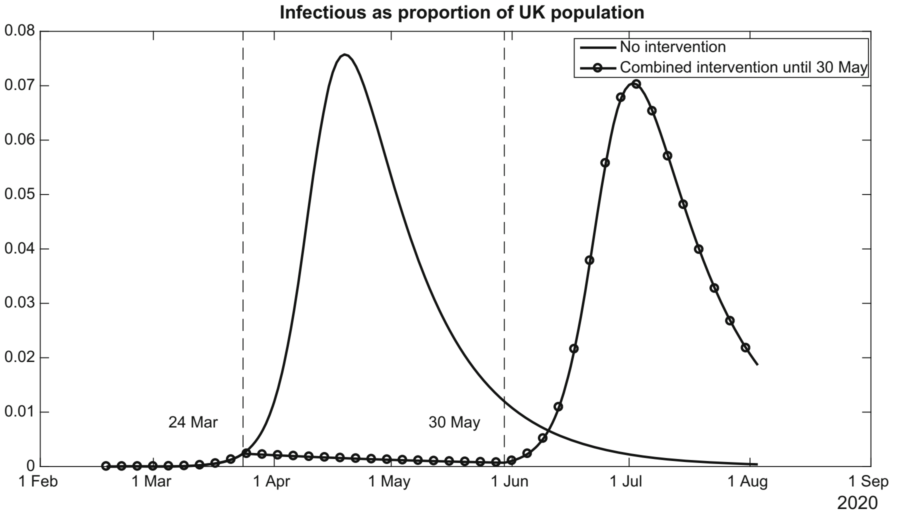

**Fig. 11** Infections (as a percentage of the UK population) under no intervention and combined intervention of $\underline{\delta} = 0.315$ and $\overline{\delta} = 0.75$ until 30 May. The vertical dash lines indicate the dates 24 March and 30 May. The plain solid line depicts infection in the absence of any intervention, while the circle line shows infections under the combined intervention of $\underline{\delta} = 0.315$ and $\overline{\delta} = 0.75$ until 30 May

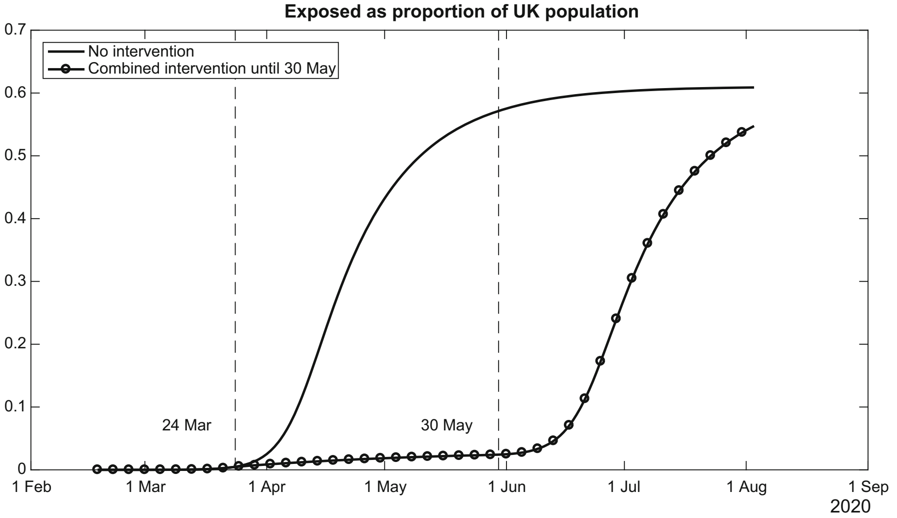

**Fig. 12** Exposure (as a percentage of the UK population) under no intervention and combined intervention of $\underline{\delta} = 0.315$ and $\overline{\delta} = 0.75$ until 30 May. The vertical dash lines indicate the dates 24 March and 30 May. The plain solid line depicts exposure in the absence of any intervention, while the circle line shows exposure under the combined intervention of $\underline{\delta} = 0.315$ and $\overline{\delta} = 0.75$ until 30 May

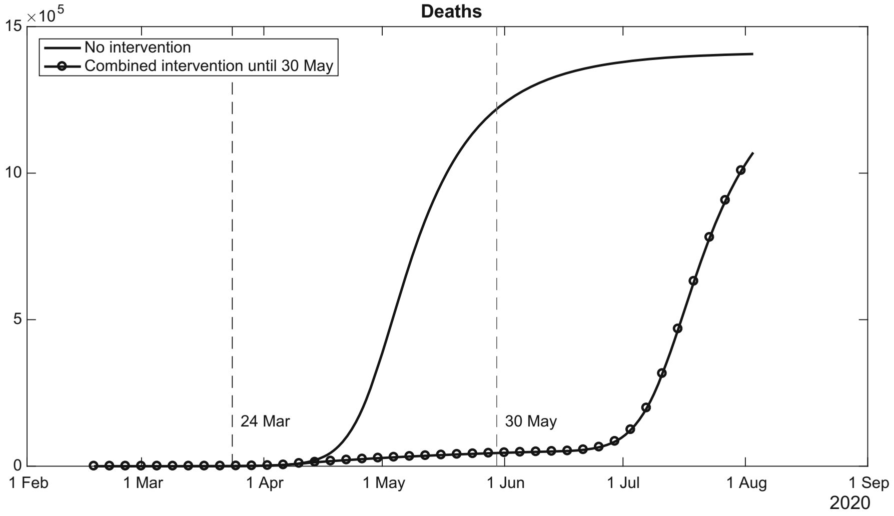

**Fig. 13** Deaths (as a percentage of the UK population) under no intervention and combined intervention of $\underline{\delta} = 0.315$ and $\overline{\delta} = 0.75$ until 30 May. The vertical dash lines indicate the dates 24 March and 30 May. The plain solid line depicts deaths in the absence of any intervention, while the circle line shows deaths under the combined intervention of $\underline{\delta} = 0.315$ and $\overline{\delta} = 0.75$ until 30 May

We show that the predicted evolution of the epidemic is significantly different when social distancing is endogenous than when the mean contact rate is assumed to be exogenous and constant. For instance, the death toll and total exposure in the calibrated model with endogenous social distancing is one third of and one quarter less than that under exogenous social distancing. Moreover, the mean contact rate, when averaged over the duration of the epidemic, is lower by about 10% than under exogenous and constant social distancing. Furthermore, under endogenous social distancing, the predicted infection peak as a proportion of the UK population is lower by 9%. Our model predicts that by the date of the first reported death around 0.009% of the UK population would have already been infected with the virus, and that the current epidemic wave in the UK would last around six months in the absence of government intervention. Our numerical analysis also suggests that the peak of the mortality rate in UK would have occurred mid April in the absence of government intervention, but with a significant death toll by the end of the epidemic.

Regarding the effects of various scenarios of government intervention on social distancing, our results suggest that the length of a lockdown that restricts the less essential activities has a significant effect on the death toll, and the "flattening of the curve", but also on the behavioural responses of low-risk individuals. These responses may lead to an after-wave. Furthermore, our numerical analysis indicates that a government intervention that restricts the more essential activities has a significant negative impact on the cumulative count of deaths, but not on the behavioural responses of low-risk individuals and the evolution of infections and herd immunity. The latter implies that such a government intervention might not be followed by an after-wave once restrictions are lifted.

Restrictions on social distancing can generate welfare gains compared to the case of no intervention. Milder but longer restrictions on less essential activities might be better in terms of welfare gains than stricter but shorter restrictions, whereas the opposite might be the case for restrictions on more essential activities. Finally, shutting down 10% more of the essential sectors might generate much larger welfare gains than shutting down 40% of the less essential sectors.

Our approach could also be used for the study of the Covid-19 epidemic in other countries where there is reliable data on deaths. The methodology in this paper could also be extended for the study of other epidemics by modifying the underlying epidemiological model appropriately.

An interesting extension of the epidemiological side of our model would be to allow for a period when infected individuals can be infectious but asymptomatic as well as for an incubation period during which infected people are asymptomatic and non-infectious, as these changes are expected to have an impact on social distancing decisions and thereby the evolution of the epidemic. Another interesting extension of our model would be to introduce heterogeneity in the time of delay from the onset of the infection to death as well as in the recovery rate from being infectious to not contributing to the spread of the infection.

In reality, individuals may not be certain about the fundamental epidemiological parameters such as the recovery and the transmission rate, or about the level of infections. In all these cases, decision-making would be taking place under uncertainty about the risks of social distancing. In such an environment, high-risk susceptible individuals might exercise a lower degree of social distancing. Loss-aversion could also make even low-risk susceptible individuals to exercise a significant degree of social distancing. Similar behavioural changes could also be observed in a model where people do not know their risk type but the population consists of two groups with different beliefs on the likelihood that they are of high or low risk. These are all interesting extensions of the economic side of our model.

**Acknowledgements** For useful comments I thank Alastair Bailey, Jagjit Chadha, Alfred Duncan, Spyros Galanis, Francesco Giovannoni, Amanda Gosling, Ilhan Guner, Miguel Leon-Ledesma, Alessandro Pavan, Flavio Toxvaerd, Yiannis Vailakis, Marios Zachariadis, and seminar participants at Kent and the Virtual Seminars in Economic Theory (VSET). I also thank the Editor and a referee for very helpful comments and suggestions. The usual disclaimer applies.

**Funding** None.

**Availability of data and material** Data on UK deaths available at https://github.com/CSSEGISandData/COVID-19.

**Code availability** https://www.dropbox.com/sh/qd8r52shhc31psf/AACBFTJ7lsrK4x-pkLgJVFTya?dl=0.

### Declarations

**Conflict of interest** None.

**Open Access** This article is licensed under a Creative Commons Attribution 4.0 International License, which permits use, sharing, adaptation, distribution and reproduction in any medium or format, as long as you give appropriate credit to the original author(s) and the source, provide a link to the Creative Commons licence, and indicate if changes were made. The images or other third party material in this article are included in the article's Creative Commons licence, unless indicated otherwise in a credit line to the material. If material is not included in the article's Creative Commons licence and your intended use is not permitted by statutory regulation or exceeds the permitted use, you will need to obtain permission directly from the copyright holder. To view a copy of this licence, visit http://creativecommons.org/licenses/by/4.0/.

## 7 Appendix

### 7.1 The SIR with heterogeneity in infection-induced mortality rates

Let $n^i$, $i = L, H$, be the probability a susceptible individual of risk-type $i$ ($L$ for low, $H$ for high) comes in contact with an infectious person. Let $I^i$ be the proportion in the population of infectious individuals of risk-type $i$ who will not die. Let $S^i$ be the proportion in the population of susceptible individuals of risk-type $i$. Let $R^i$ be the proportion in the population of exposed and non-infectious individuals of risk-type $i$ who will not die. Let $\Lambda_I$ be the proportion in the population of infectious individuals of high risk who will die (in $\psi$ periods). Let $\Lambda_R$ be the proportion in the population of exposed individuals of high risk who will die (in $\psi$ periods) but are non-infectious due to hospitalisation.

There is the standard infection flow from $S^L$ to $I^L$ at rate $n^L \beta I$ and from $S^H$ to $I^H$ at rate $(1-\theta)n^H \beta I$. These rates differ from the standard ones to accommodate for the respective contact probabilities $n^i$, $i = L, H$, and that only a fraction of high-risk individuals survive the infection. There are also the standard flows from $I^L$ to $R^L$ and from $I^H$ to $R^H$, at rate $\sigma$, of recovered individuals. In our model, however, there are additional flows. There is a flow from $S^H$ to $\Lambda_I$ of the share of the population that is of high risk, becomes infectious and will die (in $\psi$ periods), at a rate $\theta n^H \beta I$. There is also a flow from $\Lambda_I$ to $\Lambda_R$ at a rate $\sigma$; this is the share of the population that is of high risk, will die (in $\psi$ periods) and become non-infectious due to hospitalisation. The epidemic is then described by the following laws-of-motion:

$$\frac{dI^L}{dt} = n^L \beta S^L I - \sigma I^L \tag{15}$$

$$\frac{dI^H}{dt} = (1-\theta) n^H \beta S^H I - \sigma I^H \tag{16}$$

$$\frac{d\Lambda_I}{dt} = \theta n^H \beta S^H I - \sigma \Lambda_I \tag{17}$$

$$\frac{d\Lambda_R}{dt} = \sigma \Lambda_I \tag{18}$$

$$\frac{dS^L}{dt} = -n^L \beta S^L I \tag{19}$$

$$\frac{dS^H}{dt} = -n^H \beta S^H I \tag{20}$$

$$\frac{dR^L}{dt} = \sigma I^L \tag{21}$$

$$\frac{dR^H}{dt} = \sigma I^H \tag{22}$$

with initial values $I^L(t_0) = (1-\rho)I_0$, $I^H(t_0) = (1-\theta)\rho I_0$, $\Lambda_I(t_0) = \theta\rho I_0$, $\Lambda_R(t_0) = 0$, $S^L(t_0) = (1-\rho)S_0$, $S^H(t_0) = \rho S_0$, $R^L(t_0) = R^H(t_0) = 0$.

Let now $\gamma = n^L(1-\rho)+n^H\rho$, $I = I^L+I^H+\Lambda_I$, $I^L = (1-\rho)I$, $I^H = \rho(1-\theta)I$, $S^L = (1-\rho)S$, $S^H = \rho S$ $R^L = (1-\rho)R$, $R^H = \rho(1-\theta)R$, $R = R^L + R^H + \Lambda_R$, and $\Lambda = \Lambda_I + \Lambda_R$ and $Z = I + R = 1 - S$. $I$ is the proportion in the population of infectious individuals of any type, which includes those who will die (after $\psi$ periods) and are not hospitalised and hence are still infectious, $\Lambda_I$. $S$ is the proportion in the population of susceptible people of any risk type. $R$ is the proportion in the population of non-infectious individuals of any risk type, which includes those who will die (after $\psi$ periods) and are hospitalised and so are non-infectious, $\Lambda_R$. Finally, $Z$ is the proportion in the population of the exposed individuals whether they are infectious or not, which includes also those who will die (after $\psi$ periods). It follows from the above laws-of-motion that:

$$\frac{dI}{dt} = \gamma\beta SI - \sigma I \tag{23}$$

$$\frac{dS}{dt} = -\gamma\beta SI = -\frac{dZ}{dt} \tag{24}$$

$$\frac{dR}{dt} = \sigma I \tag{25}$$

with initial values $I(t_0) = I_0 \in (0, 1)$, $S(t_0) = S_0 = 1 - I_0 = 1 - Z(t_0) \in (0, 1)$ and $R(t_0) = 0$. These are as in the standard SIR model. Note also that here we have a law-of-motion for deaths:

$$\frac{d\Lambda}{dt} = \theta n^H \rho\beta SI \tag{26}$$

with $\Lambda^\psi(t_0) = \theta\rho I_0$.

### 7.2 NPVs of being at the susceptible and infectious states

Let the flow utility from being infectious and of low risk be $v_I^L$, the flow utility from being infectious and of high risk be $v_I^H$, the flow utility from being non-exposed and of low risk be $v_S^L$, the flow utility from being non-exposed and of high risk be $v_S^H$, the flow utility of being exposed and recovered and of low risk be $v_R^L$, and the flow utility of being exposed and recovered and of high risk be $v_R^L$. Assume that

$$v_S^i \geq v_R^i \geq v_I^i \quad i = L, H$$

and

$$v_j^L \geq v_j^H, \quad j = S, R, I$$

Given that Recovered is an absorbing state, we have that the NPV of being recovered and of risk-type $i = L, H$, $V_R^i$, is equal to

$$V_R^i = \frac{v_R^i}{\xi}$$

Consider next an interval $[t, t + dt)$ with $dt$ very small. We have that the NPV of being infectious and of low risk at time $t$, $V_I^L$ is equal to

$$V_I^L = v_I^L dt + [1 - \sigma dt]e^{-\xi dt} V_I^L + \sigma dt e^{-\xi dt} V_R^L$$

Using the approximation $e^y = 1 + y + \frac{y^2}{2} + \frac{y^3}{6} + \frac{y^4}{24} + \cdots + \frac{y^n}{n!} + \cdots$ we have that

$$V_I^L(1 + \xi dt) = v_I^L dt + (1 - \sigma dt)V_I^L + \sigma dt V_R^L + o^L(dt)$$

where

$$o^L(dt) = v_I^L dt \left\{\xi dt + \frac{(\xi dt)^2}{2} + \frac{(\xi dt)^3}{6} + \cdots\right\} - V_I^L \left\{\frac{(\xi dt)^2}{2} + \frac{(\xi dt)^3}{6} + \cdots\right\}$$

Canceling terms, rearranging, dividing by $dt$, then taking the limit as $dt \to 0$ and using the above equation for $V_R$, we derive that

$$V_I^L = \frac{v_I^L + \sigma \frac{v_R^L}{\xi}}{\xi + \sigma} \leq \frac{v_R^L}{\xi} = V_R^L$$

The NPV of being infectious at time $t$ and of high risk but surviving the infection, $V_I^H$, is equal to

$$V_I^H = v_I^H dt + [1 - \sigma dt]e^{-\xi dt} V_I^H + \sigma dt e^{-\xi dt} V_R^H$$

Using similar steps to the ones above, we have that

$$V_I^H = \frac{v_I^H + \sigma \frac{v_R^H}{\xi}}{\xi + \sigma} \leq \frac{v_R^H}{\xi} = V_R^H$$

Finally, let $V_D$ be the NPV of being infected in period $t'$, and of high risk and dying in period $t' + \psi$. With a constant utility flow in the first $\psi$ periods (which includes any disutility due to the anticipation of death), $V_D$ is constant.

#### 7.3 Non-cooperative social distancing by susceptible individuals

Consider an interval $[t, t + dt)$ and an individual of risk type $i = L, H$, who is susceptible at instant $t$ and chooses the probability $n^i$, with which they get in contact with each and every of the other individuals in the population, while taking as given the mortality rate for high-risk individuals, $F(\rho I)$, the transmission rate, $\beta$, and the stocks of infection $I$ and susceptibility $S$.

Recall that $n^L \beta I$ is the probability of a low-risk individual getting infected, and that $n^H \beta I$ is the probability of a high-risk individual getting infected. Recall also that a high-risk individual becomes critically ill with probability $F(\rho I)$ after being infected. Thus, $F(\rho I) n^H \beta I$ is the transition rate from the state of being susceptible and of high risk to the state of being critically ill and eventually dying. Let $W^L = V_I^L$ and $W^H = [1 - F(\rho I)] V_I^H + F(\rho I) V_D$ be the expected NPVs of low- and high-risk susceptible individuals, respectively, from being infected.

The best NPV that can be obtained at any time $t$ from being susceptible and of risk-type $i$ given the level of infection $I$ and susceptibility $S$, denoted by $V_S^i(I, S)$, is equal to

$$V_S^i(I, S) = \max_{\underline{n} \leq n \leq \overline{n}} \left\{ \left[ v_S^i - \frac{(1-n)^2}{2} \right] dt \right.$$
$$\left. + (1 - n\beta I dt)\, e^{-\xi dt} V_S^i \left( I + \frac{dI}{dt} dt, S + \frac{dS}{dt} dt \right) + n\beta I dt e^{-\xi dt} W^i \right\}$$

Note that, with $dt$ being very small, we have

$$V_S^i \left( I + \frac{dI}{dt} dt, S + \frac{dS}{dt} dt \right) = V_S^i(I, S) + \frac{\partial V_S^i(I, S)}{\partial I} \frac{dI}{dt} dt + \frac{\partial V_S^i(I, S)}{\partial S} \frac{dS}{dt} dt$$
$$= V_S^i(I, S) + \frac{\partial V_S^i(I, S)}{\partial I} I \left[ \beta \gamma S - \sigma \right] dt - \frac{\partial V_S^i(I, S)}{\partial S} \beta \gamma S I$$

Using similar steps to the ones above, we then have that

$$\xi V_S^i(I, S) = \max_{\underline{n} \leq n \leq \overline{n}} \left\{ v_S^i - \frac{(1-n)^2}{2} - n\beta I V_S^i(I, S) + n\beta I W^i \right.$$
$$\left. + \frac{\partial V_S^i(I, S)}{\partial I} I \left[ \beta \gamma S - \sigma \right] - \frac{\partial V_S^i(I, S)}{\partial S} \beta \gamma S I \right\} \quad (27)$$

where $\gamma$, $I$, $S$ and $W^i$ are taken as given.

Let $\delta = 1 - n$. It follows that social distancing of risk-type $i$ individuals given the states $S$ and $I$ solves the following problem:

$$\max_{0 \leq \delta \leq \overline{\delta}} \left\{ \delta \beta I \left[ V_S^i - W^i \right] - \frac{\delta^2}{2} \right\} \quad (28)$$

Let $\delta^i\left(V_S^i, W^i, I\right)$ denote the solution of the above maximisation problem. We then have that the social distancing in equilibrium of risk-type $i$ individuals given the states $S$ and $I$ is given by

$$\delta^i\left(V_S^i, W^i, I\right) = min\{max\left\{\beta I\left[V_S^i - W^i\right], \underline{\delta}\right\}, \overline{\delta}\}$$

and

$$\frac{dV_S^i}{dt} = \{\xi + \left[1 - \delta^i\left(V_S^i, W^i, I\right)\right]\beta I\}V_S^i - v_S^L + \frac{\left(\delta^i\left(V_S^i, W^i, I\right)\right)^2}{2}$$
$$-\left[1 - \delta^i\left(V_S^i, W^i, I\right)\right]\beta I W^i$$

where the first condition is the necessary condition of the above maximisation problem (28) and the second condition is Eq. (27) rearranged, after using the definition of $\delta^i\left(V_S^i, I\right)$ and that

$$\frac{dV_S^L}{dt} = \frac{\partial V_S^i(I,S)}{\partial I}\frac{dI}{dt} + \frac{\partial V_S^i(I,S)}{\partial S}\frac{dS}{dt} = \beta\gamma SI\frac{\partial V_S^i(I,S)}{\partial I}I\left[\beta\gamma S - \sigma\right] - \frac{\partial V_S^i(I,S)}{\partial S}\beta\gamma SI$$

We can then derive the problem of susceptible individuals as stated in the main text by setting:

$$C_L \equiv K_L = V_S^L - W^L = V_S^L - V_I^L = V_S^L - \frac{v_I^L + \sigma\frac{v_R^L}{\xi}}{\xi + \sigma}$$
$$K_H = V_S^H - V_I^H = V_S^H - \frac{v_I^H + \sigma\frac{v_R^H}{\xi}}{\xi + \sigma}$$

and

$$\overline{K} = V_I^H - V_D = \frac{v_I^H + \sigma\frac{v_R^H}{\xi}}{\xi + \sigma} - V_D$$

and so

$$C_H \equiv K_H + F(\rho I)\overline{K} = V_S^H - W^H = V_S^H - V_I^H + F(\rho I)\left[V_I^H - V_D\right]$$

Note that $\overline{K} > 0$ if $V_D$ is small enough, which is assumed to be the case. We also assume that $v_I^L + \sigma\frac{v_R^L}{\xi}$ (for given $\sigma$ and $\xi$) is sufficiently higher than $v_I^H + \sigma\frac{v_R^H}{\xi}$ so that $K_L < K_H$.

Finally, note that social distancing by high-risk individuals depends on the mortality rate, which depends on level of infection. After using the above definitions, the equilibrium social distancing distancing $\left(\delta_L^*\left(K_L, I\right), \delta_H^*\left(K_H, I\right)\right)$ is given by:

$$\delta_L^*\left(K_L, I\right) = min\{max\{\beta I K_L, \underline{\delta}\}, \overline{\delta}\}$$

$$\delta_H^*\left(K_H, I\right) = min\{max\{\beta I \left[K_H + F\left(\rho I\right)\overline{K}\right], \underline{\delta}\}, \overline{\delta}\}$$

where $K_L$ and $K_H$ evolve over time according to the following system of ODEs (given a path of $I$).

$$\begin{aligned}\frac{\partial K_L}{\partial t} = \frac{dV_S^L}{dt} &= \{\xi + \left[1 - \delta_L^*\left(K_L, I\right)\right]\beta I\}\left[K_L + V_I^L\right] - v_S^L + \frac{\left(\delta_L^*\left(K_L, I\right)\right)^2}{2} \\ &\quad - \left[1 - \delta_L^*\left(K_L, I\right)\right]\beta I V_I^L \\ &= \xi\left[K_L + V_I^L\right] + \left[1 - \delta_L^*\left(K_L, I\right)\right]\beta I K_L - v_S^L + \frac{\left(\delta_L^*\left(K_L, I\right)\right)^2}{2}\end{aligned}$$

and

$$\begin{aligned}\frac{\partial K_H}{\partial t} = \frac{dV_S^H}{dt} &= \{\xi + \left[1 - \delta_H^*\left(K_H, I\right)\right]\beta I\}\left[K_H + V_I^H\right] - v_S^H + \frac{\left(\delta_H^*\left(K_H, I\right)\right)^2}{2} \\ &\quad - \left[1 - \delta_H^*\left(K_H, I\right)\right]\beta I\left[V_I^H - F\left(\rho I\right)\overline{K}\right] \\ &= \xi\left[K_H + V_I^H\right] + \left[1 - \delta_H^*\left(K_H, I\right)\right]\beta I\left[K_H + F\left(\rho I\right)\overline{K}\right] - v_S^H + \frac{\left(\delta_H^*\left(K_H, I\right)\right)^2}{2}\end{aligned}$$

The above ODEs become the laws-of-motion stated in the main text after setting $\phi^i = \xi V_I^i - v_S^i, i = L, H$. Note that $\phi^i \leq 0$.

For the calibrations, we set $v_S^L = v_S^H = v_S$ and normalise units by setting $v_S = 0$. This implies that $v_I^i \leq v_R^i \leq v_S \leq 0$ and

$$\phi^H = \frac{\xi v_I^H + \sigma v_R^H}{\xi + \sigma} \leq \phi^L = \frac{\xi v_I^L + \sigma v_R^L}{\xi + \sigma} \leq v_S = 0$$

Note also that

$$V_D = \frac{\phi^H}{\xi} - \overline{K} < 0$$

Finally note that with logarithmic utility, and denoting with $c_j^i$ , $i = L, H$, $j = S, I, R$, the state- and risk-type dependent consumption, we can define consumption-equivalent units $c^i$, $i = L, H$, and $c_D$, and a proportionate payment stream $p$ such that $\phi^i = log\left(\left(c_I^i\right)^{\frac{\xi}{\xi+\sigma}}\left(c_R^i\right)^{\frac{\sigma}{\xi+\sigma}}\right) = log\left(c^i\right)$ and $V_D = \frac{log(c_D)}{\xi} = \frac{log\left(c^H(1-p)\right)}{\xi}$. Therefore, we can express $\overline{K}$ as

$$\overline{K} = \frac{log\left(c^H\right) - log\left(c^H\left(1-p\right)\right)}{\xi} = \frac{log\left(1\right) - log\left(1-p\right)}{\xi} = \frac{-log\left(1-p\right)}{\xi}$$

#### 7.4 Optimal social distancing of susceptible individuals

Here we solve the problem of finding the optimal from the point of view of the society social distancing of susceptible individuals from a period $t_g$ onwards, while taking into account the implications for aggregate infections. Specifically, we find the social distancing profile of susceptible individuals that maximises total welfare in the economy. To ease exposition, we assume without loss of generality that infectious individuals do not exercise any social distancing.

Recall that $n^L \beta I$ is the probability of a low-risk individual getting infected, and that $n^H \beta I$ is the probability of a high-risk individual getting infected. Recall also that a high-risk individual becomes critically ill with probability $F(\rho I)$ after being infected. Thus, $F(\rho I) n^H \beta I$ is the transition rate from the state of being susceptible and of high risk to the state of being critically ill and eventually dying. Recall also from the previous subsection in this appendix the definition that $W^L = V_I^L$ and $W^H = [1 - F(\rho I)]\, V_I^H + F(\rho I) V_D$.

Let also $V_R = (1-\rho) V_R^H + \rho V_R^H$ and $V_I = (1-\rho) V_I^H + \rho V_I^H$. Using the results in the previous subsection in this appendix, we have that $V_R - V_I \geq 0$ and that $V_R$ and $V_I$ are constants. Let also $\gamma(n^L, n^H) = (1-\rho) n^L + \rho n^H$. ,Given the constants $V_R$ and $V_I$ and the levels of infection and susceptibility $I$and $S$, define here with some abuse of notation the NPV of susceptible people that is obtained by optimal social distancing.

Given the levels of infection $I$ and susceptibility $S$ at instant $t \geq t_g$, the government chooses the probabilities $(n^L, n^H)$, with which low-risk and high-risk susceptible individuals, respectively, get in contact with each and every of the other individuals in the population, while taking into account how these probabilities affect the mortality rate for high-risk individuals, $F(\rho I)$, and the evolution of the population shares of infectious and susceptible people. With some abuse of notation, let $\widetilde{V}_S^i(n^L, n^H, S, I)$ be the NPV of a susceptible individual of risk type $i = L, H$, given the levels of infection $I$ and susceptibility $S$, and the social distancing profile $(n^L, n^H)$ at instant $t$. We then have that optimal distancing solves the following problem:

$$\max_{1-\overline{\delta} \leq n^L, n^H \leq 1-\underline{\delta}} \left\{ [1 - S - I]\, V_R + I V_I + S \left[ (1-\rho) \widetilde{V}_S^L(n^L, n^H, S, I) + \rho \widetilde{V}_S^H(n^L, n^H, S, I) \right] \right\}$$

Clearly, then, optimal social distancing solves also the following problem:

$$\max_{1-\overline{\delta} \leq n^L, n^H \leq 1-\underline{\delta}} \left\{ (1-\rho) \widetilde{V}_S^L(n^L, n^H, S, I) + \rho \widetilde{V}_S^H(n^L, n^H, S, I) \right\}$$

Let $V_S^i(I, S)$ be the NPV for a susceptible individual of risk type $i = L, H$, evaluated at the optimum social distancing profile. Let $V_S(I, S) = (1-\rho) V_S^L(I, S) + \rho V_S^L(I, S)$, and note that $V_S(I, S)$ is the value function of the above maximisation problem.

Consider now an interval $[t, t + dt)$, with $t \geq t_g$ and $dt$ very small. We thus have that

$$\begin{aligned}
&V_S(I, S) \\
&= \max_{1-\overline{\delta} \leq n^L, n^H \leq 1-\underline{\delta}} \Bigg\{(1-\rho)\left\{\left[v_S^L - \frac{(1-n^L)^2}{2}\right]dt + n^L\beta I dt e^{-\xi dt} V_I^L\right. \\
&\quad \left. + (1 - n^L\beta I dt)e^{-\xi dt} V_S^L \left(I + \frac{dI}{dt}dt, S + \frac{dS}{dt}dt\right)\right\} \\
&\quad + \rho\left\{\left[v_S^H - \frac{(1-n^H)^2}{2}\right]dt + n^H\beta I dt e^{-\xi dt}\left[V_I^H - F(\rho I)\left[V_I^H - V_D\right]\right]\right. \\
&\quad \left. + (1 - n^H\beta I dt)e^{-\xi dt} V_S^H (I + \frac{dI}{dt}dt, S + \frac{dS}{dt}dt)\right\}\Bigg\}
\end{aligned}$$

given the laws-of-motion $\frac{dI}{dt}, \frac{dS}{dt}$. Note that, with $dt$ being very small, we have

$$\begin{aligned}
V_S^i\left(I + \frac{dI}{dt}dt, S + \frac{dS}{dt}dt\right) &= V_S^i(I, S) + \frac{\partial V_S^i(I, S)}{\partial I} I\left[\beta\gamma(n^L, n^H)S - \sigma\right]dt \\
&\quad + \frac{\partial V_S^i(I, S)}{\partial S}\beta\gamma(n^L, n^H)SI dt
\end{aligned}$$

Using similar steps to the ones in the previous subsection in this appendix, we have that

$$\begin{aligned}
&\xi V_S(I, S) \\
&= \max_{1-\overline{\delta} \leq n^L, n^H \leq 1-\underline{\delta}} \Bigg\{(1-\rho)\left\{\left[v_S^L - \frac{(1-n^L)^2}{2}\right] + n^L\beta I\left[V_I^L - V_S^L(I, S)\right]\right. \\
&\quad \left. + \frac{\partial V_S^L(I, S)}{\partial I} I\left[\beta\gamma(n^L, n^H)S - \sigma\right] + \frac{\partial V_S^L(I, S)}{\partial S}\beta\gamma(n^L, n^H)SI\right\} \\
&\quad + \rho\left\{\left[v_S^H - \frac{(1-n^H)^2}{2}\right] + n^H\beta I\left[\left[V_I^H - V_S^H(I, S)\right] - F(\rho I)\left[V_I^H - V_D\right]\right]\right. \\
&\quad \left. + \frac{\partial V_S^H(I, S)}{\partial I} I\left[\beta\gamma(n^L, n^H)S - \sigma\right] + \frac{\partial V_S^{iH}(I, S)}{\partial S}\beta\gamma(n^L, n^H)SI\right\}\Bigg\}
\end{aligned}$$

Clearly then, after letting $\delta_i = 1 - n^i$, $i = L, H$, and recalling the definition of $\overline{K}$ from the previous subsection in this appendix, the government solves, in effect, the following problem:

$$\max_{1-\bar{\delta}\leq n^L,n^H\leq 1-\underline{\delta}} \left\{(1-\rho)\left\{\delta_L \beta I\left[V_S^L(I,S)-V_I^L\right]-\frac{(\delta_L)^2}{2}\right\}\right.$$
$$+\rho\left\{\beta I\left\{\delta_H\left[V_S^H(I,S)-V_I^H\right]-(1-\delta_H)F(\rho I)\overline{K}\right\}-\frac{(\delta_H)^2}{2}\right\}$$
$$+\beta\gamma((1-\delta_L),(1-\delta_H))SI\left\{(1-\rho)\left[\frac{\partial V_S^L(I,S)}{\partial I}+\frac{\partial V_S^L(I,S)}{\partial S}\right]\right.$$
$$\left.\left.+\rho\left[\frac{\partial V_S^H(I,S)}{\partial I}+\frac{\partial V_S^H(I,S)}{\partial S}\right]\right\}\right\}$$

Therefore, compared to the problem solved by susceptible individuals, the government internalises the externalities generated by the social distancing of each risk-type through the impact of social distancing on the mean contact rate and thereby on the mortality rate and the evolution of the epidemic.

#### 7.4.1 Solving for the optimal social distancing

Let

$$DV_S(I,S)\equiv(1-\rho)\left[\frac{\partial V_S^L(I,S)}{\partial I}+\frac{\partial V_S^L(I,S)}{\partial S}\right]+\rho\left[\frac{\partial V_S^H(I,S)}{\partial I}+\frac{\partial V_S^H(I,S)}{\partial S}\right]$$

The necessary conditions of the above maximisation problem are:

$$\beta I\left[V_S^L(I,S)-V_I^L\right]-\delta_L=\beta SIDV_S(I,S)$$

and

$$\beta I\left\{\left[V_S^H(I,S)-V_I^H\right]+F(\beta\gamma(n^L,n^H)SI)\overline{K}\right\}-\delta_H=\beta SIDV_S(I,S)$$

Note that individuals do not internalise the effect of their decisions on the evolution of the epidemic either. An increase in current social distancing (of any given individual) reduces the new infections and thereby it reduces the future population share of infectious people while it increases the future population share of the susceptible part of the population. These changes have the following implications.

First, a decrease in the level of infection decreases the expected net present value of the cost of infection. It also decreases the new infections, and thereby the future level of infection and hence the future expected net present values of the cost of infection, and so on. These effects push towards $DV_S(I,S)>0$. However, note also that a decrease in the level of infection also decreases mortality rates and therefore the expected cost from dying. This effect pushes towards $DV_S(I,S)<0$. Second, an increase in the level of susceptibility increases the new infections and hence the future level of infection and thereby the expected net present value of the cost of infection in the future. These effects push towards $DV_S(I, S) > 0$. Therefore, the overall welfare effect of changes in the evolution of the epidemic and thereby the associated externality of social distancing, as captured by the term $DV_S(I, S)$, cannot be signed without further restrictions on the primitives of the model.

We assume that $\overline{K}$ is high enough to ensure that the positive externality via the effect of social distancing on the mortality rate ($\psi$ periods ahead) dominates, i.e.

$$\beta SIDV_S(I, S) < 0$$

and so the overall externality generated by social distancing is positive. It follows directly (from the first two optimality conditions) that the optimal social distancing is (weakly) higher than private choices,[41] with optimal social distancing from high-risk individuals being higher than that from low-risk individuals.
